# Antidepressants fluoxetine and amitriptyline induce alterations in intestinal microbiota and gut microbiome function in rats exposed to chronic unpredictable mild stress

**DOI:** 10.1038/s41398-021-01254-5

**Published:** 2021-02-18

**Authors:** Weijie Zhang, Wan Qu, Hua Wang, He Yan

**Affiliations:** 1 College of Food Science and Engineering, South China University of Technology, 510000 Guangzhou, China; 2 Department of Food Science and Technology, The Ohio State University, Columbus, OH USA; 3 Research Institute for Food Nutrition and Human Health, 510000 Guangzhou, China

**Keywords:** Prognostic markers, Pharmacogenomics

## Abstract

Antidepressant medications are known to modulate the central nervous system, and gut microbiota can play a role in depression via microbiota–gut–brain axis. But the impact of antidepressants on gut microbiota function and composition remains poorly understood. Thus this study assessed the effect of serotonin reuptake inhibitor antidepressant fluoxetine (Flu) and tricyclic antidepressant amitriptyline (Ami) administration on gut microbiota composition, diversity, and species abundance, along with microbial function in a chronic unpredictable mild stress (CUMS)-induced depression rat model. Oral administration of Ami and Flu significantly altered the overall gut microbiota profile of CUMS-induced rats, as assessed using the permutational multivariate analysis of variance test. At the phylum level, 6-week of antidepressant treatment led to a decreased Firmicutes/Bacteroidetes ratio due to an enhanced Bacteroidetes and reduced Firmicutes relative abundance. Flu was more potent than Ami at altering the Firmicutes and Bacteroidetes levels in the CUMS rats. At the family level, both antidepressants significantly increased the abundance of Porphyromonadaceae. However, an increased Bacteroidaceae level was significantly associated with Ami, not Flu treatment. Furthermore, at the genus level, an increase in the relative abundance of *Parabacteroides*, *Butyricimonas*, and *Alistipes* was observed following Ami and Flu treatment. Subsequent metagenomics and bioinformatics analysis further indicated that Ami and Flu likely also modulated metabolic pathways, such as those involved in carbohydrate metabolism, membrane transport, and signal transduction. Additionally, both antidepressants affected antibiotic resistome, such as for aminoglycoside (*aph3iii*A), multidrug (*mdt*K, *mdt*P, *mdt*H, *mdt*G, *acr*A), and tetracycline (*tet*M) resistance in CUMS rats. These data clearly illustrated the direct impact of oral administration of Flu and Ami on the gut microbiome, thus set up the foundation to reveal more insights on the therapeutic function of the antidepressants and their overall contribution to host health.

## Background

Depression, a widespread and debilitating mental disorder, is characterised by low sensitivity, mood, sleeplessness, and loss of interest in enjoyable activities^1,2^. The complex aetiology involves dysregulated neuroendocrine^3^, neuroimmune^4^, metabolic^5^, and neurotransmitter systems^6^. According to the World Health Organisation, depression affects ~350 million people worldwide^7^ and has become one of the most attention-worthy disorders owing to the high rates of morbidity and mortality^8–10^. A number of therapies have been applied clinically for the treatment of depression, including drug therapy, psychotherapy, physical therapy, exercise therapy, and acupuncture^11^. In particular, antidepressants represent a major and widely used means of treating depression and are globally available^12^.

The gut microbiota–brain axis is a complex multi-organ bidirectional signalling system between intestinal flora and the brain, which has a crucial role in host physiology, homoeostasis, development, and metabolism^13,14^. Recent data have illustrated that gut microbiota is strongly associated with the pathology of several mental disorders, including depression and anxiety^15–17^. For example, a clinical study on the gut microbiota profiles of 37 depressed patients and 18 non-depressed controls found that the operational taxonomic units (OTUs) of the order Bacteroidales were overrepresented, while the family Lachnospiraceae was underrepresented in patients suffering from depression^15^. Moreover, data from preclinical studies emerged over the past two decades have also suggested the association between psychological stress and gut microbiota dysbiosis. For instance, using a rhesus macaque model, Bailey and Coe^18^ illustrated that alterations in gut microbiota composition correlate with anxiety-related behaviours and serum stress hormone levels related to early-life stress. Another recent study showed that the transfer of fecal microbiota from humans with depression into microbiota-depleted rats or mice induced a depressive-like phenotype, indicating that gut microbiota may play a key role in the onset of depressive behaviour^19^. In addition to the correlation between disease and gut microbiota, the complex relationship between drugs and gut microbiota has also attracted extensive attention. For example, the use of antibiotics that disturb the gastrointestinal flora is associated with clinical symptoms such as diarrhoea^20^ and inflammatory bowel diseases in childhood^21^. Antibiotic-induced microbiome-depleted in butyrate-producing microbiota is associated with type 2 diabetes^22^. Furthermore, recent studies have indicated that certain common non-antibiotic drugs also impact gut microbiome^23,24^. However, only very limited antidepressants have been evaluated for their effects on gut microbiota. For instance, Yang^25^ reported that the antidepressant effects of two ketamine enantiomers in a chronic social defeat stress (CSDS) model may be partially mediated by the restoration of the gut microbiota. Moreover, daily oral administration of fluoxetine to healthy male mice significantly decreased the abundance of beneficial *Lactobacillus johnsonii* species, suggesting that the negative clinical effects of fluoxetine may be caused by alterations in the gut microbiota^26^. Similarly, treatment with lithium, valproate or aripiprazole also increases the abundance of several species, including those belonging to the *Clostridium, Peptoclostridium, Intestinibacter,* and Christenellaceae in healthy adult rats^27^.

Fluoxetine hydrochloride (Flu), a type of selective serotonin reuptake inhibitor antidepressant (SSRI) discovered in the 1970s, maintains serotonin levels in the synaptic cleft primarily by inhibiting serotonin reuptake^28^. Globally, it is among the most prescribed pharmaceutical active substances and is generally prescribed to patients diagnosed with clinical depression, obsessive-compulsive disorder, panic disorder, social phobia, or attention-deficit disorder^29^. Meanwhile, a representative tricyclic antidepressant, amitriptyline (Ami) has been prescribed for decades to treat depression, with a half-life of 20 h^30^. The antidepressant mode of action of Ami is generally associated with blockade of serotonin and norepinephrine uptake in the central nervous system^31^. Ami also possesses a multiplicity of other distinct pharmacological activities, such as antagonist actions at histamine, muscarinic, α1-adrenergic, and serotonin receptors at nanomolar concentrations, as well as at several ion channels at micromolar concentrations^32^. Together, Flu and Ami constitute the mainstream antidepressants used for treating depression. However, the complex pathogenesis associated with depression is not fully understood, thus impeding effective therapeutics for this disease. Moreover, unintended, including serious adverse consequences of antidepressants have been reported, while the mechanisms remain to be elucidated^33^. Despite several attempts to elucidate the effects of antidepressants on the intestinal microbiota^25,27^, the influence of Flu or Ami on the structure and function of intestinal microorganisms under conditions of depression has not been fully characterised.

Meanwhile, although antidepressant drugs are widely known for their neuroprotective properties, their antimicrobial effects in vitro when used as psychotropic medications have been reported in several studies^34^. For instance, the new generation antidepressant drugs, such as sertraline, fluoxetine, amitriptyline, escitalopram, as well as older drugs, such as tranylcypromine and imipramine, possess antimicrobial effects in vitro^35,36^. However, antimicrobial resistance is primarily selected upon antimicrobial use^37,38^. The potential contribution by antidepressant drugs to the rise of antibiotic resistance in gut microbiota in vivo is so far underestimated. An improved understanding on the impact of antidepressants on gut microbiome becomes essential for proper evaluation of therapeutic options.

Therefore, the objective of the study was to investigate the impact of antidepressant treatment on the gut microbiome of chronic unpredictable mild stress (CUMS) rats, an animal model of depression. In addition to the commonly used 16S rRNA gene sequencing, we also used shotgun metagenomics analyses for the Kyoto Encyclopaedia of Genes and Genomes (KEGG) metabolic pathways and antibiotic resistance genes (ARGs), to compare the functional profiles. The data not only contribute to the knowledge of gut impacting drugs but also have direct clinical implications.

## Methods

### Animal model and experimental timeline

The animal protocol for the study was approved by Institutional Animal Care and the Committee on the Ethics of Animal Experiments of South China Agricultural University (Permit Number 2017-B017). Pathogen-free adult male Sprague–Dawley rats (180–220 g) were obtained from Guangdong Medical Laboratory Animal Center (Guangzhou, China). Overall, the animal study lasted for 15 weeks. All rats were acclimatised for 1 week to the laboratory environment (12 h light/dark cycle, 25 ± 1 °C, and 55–65% relative humidity), with ad libitum access to food and water; each rat was housed per cage. The experimental timeline is presented in Supplementary Fig. 1. Following acclimatisation, 48 rats were randomly divided into two groups: healthy control (HC; *n* = 12) and CUMS (*n* = 36), with all rats being housed independently. With the exception of HC rats, all were subjected to eight different chronic unpredictable mild stimuli for 14 weeks, including 8 weeks of depression model development and a 6-week treatment period. The CUMS-resistant rats were screened using behaviour tests (sucrose preference test (SPT), open field test (OFT) and light/dark test (LDT)) after 8 weeks of exposure to CUMS. To ensure adequate statistical power, the remaining rats were randomly divided into three groups according to body weight as follows: model (CUMS, *n* = 6), Ami treatment (Ami, *n* = 6), and Flu treatment (Flu, *n* = 7) groups. The Flu used in this study was purchased from LILLY (Eli Lilly and Company, IN, USA). Amitriptyline was purchased from Dongting (Hunan Dongting Pharmaceutical, Hunan, China). The antidepressant groups were administered with Ami (25 mg/kg/d) or Flu (12 mg/kg/d) for 6 weeks by oral gavage, whereas the HC and CUMS rats were administered with equal volumes of sterile water. SPT was performed during weeks 0, 9, and 15. Behaviour tests (OFT, LDT) were performed pre- and post-Ami and Flu treatment. Faecal samples were collected at weeks 9 and 15. At week 9, using 16 S rRNA gene sequencing, faecal samples from randomly chosen CUMS-induced (*n* = 12) and HC (*n* = 12) rats were collected and subjected to 16S rRNA gene sequencing analysis. In addition, faecal samples of 3 individual rats randomly chosen from each group (HC: *n* = 3; CUMS: *n* = 3; Ami: *n* = 3; Flu: *n* = 3) were subjected to metagenome analysis at week 15. At the end of the experiment, the rats were fasted for 12 h, anaesthetised using 6% (v/v) chloral hydrate, and euthanised. In these studies, no blinding was done.

### Chronic unpredictable mild stress

The CUMS procedures were performed as previously described^39,40^. The protocol comprised eight stressors: food deprivation for 24 h, water deprivation for 24 h, flash stimulation (150 flashes/min for 5 min), cage tilting at 45° for 24 h, overnight illumination for 8 h, wet cage environment (200 mL water added to sawdust bedding) for 24 h, tail suspension for 5 min, 60 °C heat stimulation for 6 min, and nipped tail for 5 min. Stressors were applied at least 13 times and without repetitive stressors in two consecutive days. To prevent being influenced by the CUMS rats, the HC rats were housed in an adjacent room and had no contact with the model animals. The model rats were authenticated by behaviour tests upon completion of the model development period.

### Behavioural testing

The body weights of all rats were recorded each week. Anxiety-like and depression-like behaviours were examined using SPT, OFT, and LDT. In SPT, rats were trained to adapt to a sucrose solution (1%, w/v): two bottles of sucrose solution were placed in each cage for 24 h, subsequently, each bottle of sucrose solution was replaced with water for 24 h. After adaptation, the mice were deprived of water and food for 24 h. The rats were then given ad libitum access to water and sucrose solution for 4 h, after which the remaining volume of water and sucrose solution were measured. Sucrose preference was calculated using the formula as follows: sucrose preference = sucrose consumption/ (water and sucrose consumption) × 100%^41^. In OFT, an open field consisting of a black square (80 × 80 × 60 cm) was divided into 16 equal squares. Each rat was placed in the centre of an open arena and rat behaviour was recorded for 5 min. Prior to the start of the test, 30 s was set for adaptation. The total number of crossings and rearings was recorded^42^. In LDT, each rat was placed in the centre of an apparatus (40 × 30 × 35 cm) containing two chambers of equal size, one bright and the other dark. Total time spent in the dark zone was recorded for 5 min^43^.

### Gut microbiota profiling by 16S rRNA gene sequencing

To profile the microbial composition, faecal samples were subjected to total genome DNA extraction using the QIAamp DNA Stool Mini Kit following the manufacturer’s instruction (Qiagen, Venlo, The Netherlands). The V4–V5 region of 16S rRNA genes of the samples was amplified by polymerase chain reaction (PCR) (98 °C for 60 s, followed by 30 cycles at 98 °C for 10 s, 50 °C for 30 s, 72 °C for 60 s, and 72 °C for 5 min), using primers 515 F 5′-GTGCCAGCMGCCGCGGTAA-3′ and 907 R 5′-CCGTCAATTCCTTTGAGTTT-3′. The sequencing libraries of the V4–V5 region of the 16 S rRNA genes were generated using the TruSeq® DNA PCR-Free Sample Preparation Kit (Illumina, San Diego, CA, USA) following the manufacturer’s instruction, and index codes were added. The libraries were sequenced using an Illumina HiSeq 2500 platform. QIIME software (Version 1.7.0)^44^ was used to analyse alpha- (within samples) and beta- (among samples) diversity. Reads were first filtered by QIIME quality filters and chimera sequences were removed using the UCHIME algorithm. The filtered sequences were then clustered into OTUs according to representative sequence using UPARSE software^45^ (Version 7.0.1001) and classified against the Greengenes database^46^ with a threshold of 97% sequence similarity. Alpha-diversity was applied toward analysing the complexity of species diversity for a sample through four indices including observed-species, Chao 1, Simpson, and Shannon. Principal-coordinate analysis (PCoA) based on weighted and unweighted UniFrac metrics was used to assess the variation of bacterial composition among different groups and phases. These analyses were performed using the free online Majorbio I-Sanger Cloud Platform (https://cloud.majorbio.com/). The 16S rRNA sequence data for representative samples were deposited in the Sequence Read Archive (SRA) database (http://www.ncbi.nlm.nih.gov/Traces/sra)^47^.

### Shotgun metagenomics analysis of faecal samples

To explore the microbial metabolic function and ARGs of the rat faecal microbiota, DNA extracts of the representative samples were further subjected to shotgun metagenomics sequencing analysis on an Illumina HiSeq 4000 platform using the HiSeq 4000 PE Cluster Kit and HiSeq 4000 SBS Kits. Open reading frames (ORFs) predicted from all samples were merged and aligned to each other. Gene pairs with >95% identity (no gap allowed) and aligned reads covering over 90% of the shorter reads were grouped together. The longest ORF in each group was used to represent the group; while, the other ORFs of the group were regarded as redundant sequences. ORFs with a length less than 100 bp were subsequently filtered out. Based on this reference gene set, taxonomic assignment and functional annotation were further conducted using the latest version (Version 2.2.28+) of the KEGG database^48^ and Comprehensive Antibiotic Resistance Database^49^. The metagenomics datasets were deposited into the SRA database.

### Network analysis

To investigate co-occurrence patterns of microbial community and ARGs, correlation matrices were constructed by calculating each pairwise Spearman’s rank correlation. A correlation between any two items was considered statistically robust if the Spearman’s correlation coefficient (*ρ*) was >0.8 and the *P*-value was <0.01^50^. The resulting correlation matrices were translated into an association network using Cytoscape v3. 7. 1^51^.

### Statistical analysis

In this study, 2,127,824 high quality-filtered and chimera-checked sequences were generated, with an average length of 372.38 bp across all samples. The mean number of reads per sample was 68,639, ranging from 58,524 to 74,062 reads. A total of 702 OTUs (97% sequence similarity) were detected among all samples. Based on relative abundance, the taxonomic analysis revealed 16 bacteria phyla, 30 classes, 49 orders, 88 families, and 194 genera across all samples. OTUs that reached 97% similarity were used for alpha-diversity estimations, which included observed OTUs (Sobs), diversity (Shannon and Simpson indices), richness (Chao I), coverage (Good’s coverage), and rarefaction curve analysis using Mothur^52^ (Version 1.30.23). Results on behaviour assessments (body weight, sucrose preference ratio, numbers of crossings and rearings, time spent in the dark zone) were compared among groups using one-way analysis of variance (ANOVA) followed by the least significant difference test using Statistical Package for Social Science programme (SPSS 22.0, Armonk, NY, USA). PCoA based on weighted and unweighted UniFrac metrics was used to assess the variation of bacterial composition among different groups and different phases. The relative abundance of faecal microbiota, ARGs, and KOs in the four groups was compared using the Kruskal–Wallis H test with Tukey’s posthoc tests was used in the case of pairwise comparison. Moreover, LEfSe analysis combining the Kruskal–Wallis test with linear discriminant analysis was used to identify the differential KEGG pathway representation between faecal microbiomes of the two groups. A threshold value >2 was used as the cut off value for statistical significance based on a *P-*value of 0.05.

## Results

### Antidepressants improved CUMS-induced depressive and anxiety-like behaviours

**Fig. 1:**
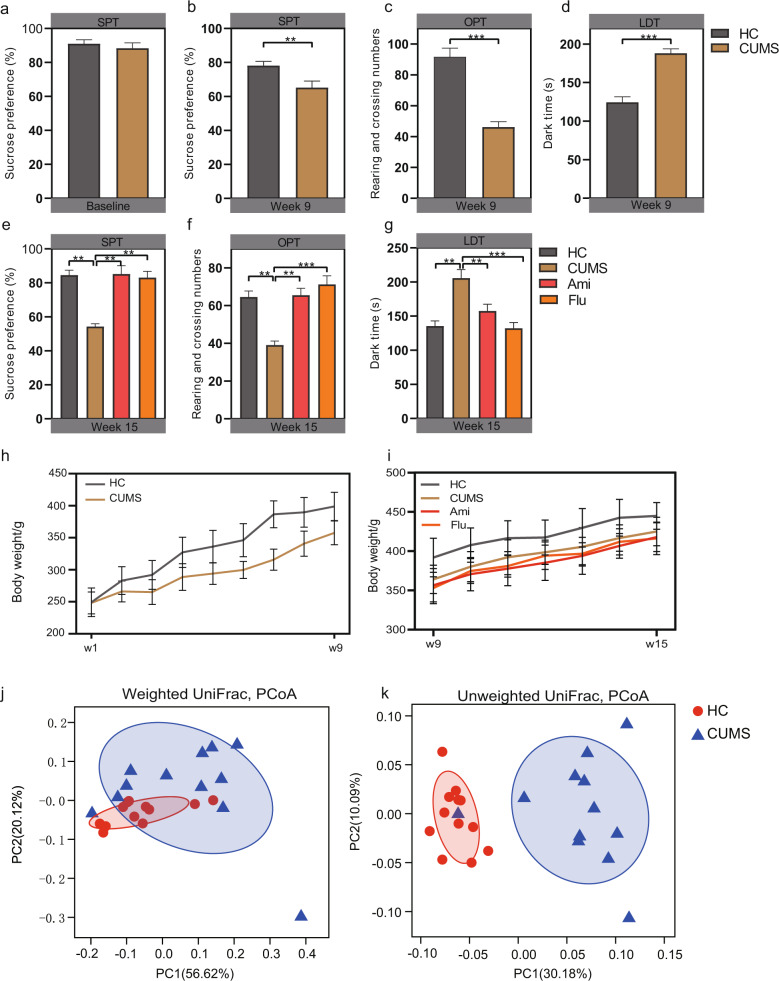
Depression-like behaviours and PCoA analysis of gut bacterium data at week 9 for evaluation of the CUMS modelling and effectiveness of antidepressant treatment. **a** Sucrose preference test at baseline. Sucrose preference (**b**), open filed test (**c**) and light/dark test (**d**) performed after 8-week CUMS exposure. Sucrose preference test (**e**), open filed test (**f**) and light/dark test (**g**) performed after six-week antidepressants treatment. Changes in body weight from **h** weeks 1–9 and **i** weeks 9–15 in HC and CUMS groups. PCoA of bacterial beta-diversity based on the **j** weighted and **k** unweighted UniFrac distances between CUMS rats and healthy controls at week 9.

As illustrated in Fig. 1a, no statistically significant differences were observed in the sucrose preference ratio among the rats during the adaptation period. After 8 weeks of model development, one-way ANOVA revealed that the results of the SPT and anxiety-like behaviour tests (e.g., sucrose preference ratio, open field rearing and crossing numbers, and total time spent in the dark zone) of the CUMS-induced rats significantly differed from those in the HC group (Fig. 1b–d). It is worth noting that using hierarchical cluster analysis, after 8-week exposure to CUMS, a small subpopulation of the rats did not express depression-like symptoms. Sequencing data showed that faecal microbiota composition of the rats without (CUMS-resistant) and with depression-like symptoms were clearly separated into two subgroups (Supplementary Fig. 2), indicating a strong association between gut microbiota composition and depression-like behaviours. The CUMS-resistant mice were then removed from the rest of the studies. Furthermore, as illustrated in Fig. 1h, the CUMS-induced rats weighed less than the HC rats. These data suggested that the CUMS-induced depressive model was successfully established. Notably, PCoA plots of the 16S rRNA gene sequence data based on weighted and unweighted UniFrac metrics showed that the bacterial microbiota of HC was clustered and separated from those of established CUMS rats at week 9 (*P* < 0.05, permutational multivariate analysis of variance (PERMANOVA); Fig. 1j, k and Supplementary Table 1). After 6 weeks of treatment with Ami or Flu, one-way ANOVA results suggested a reversal of the depression and anxiety behaviours of the CUMS-induced rats, including the recovery of sucrose preference ratio, improved activity rates, and significantly decreased time spent in the dark zone (Fig. 1e–g). However, there was no significant change in the weight of the rats treated with antidepressants (Fig. 1i).

### Oral antidepressants alter faecal microbiota alpha and beta-diversity

**Fig. 2:**
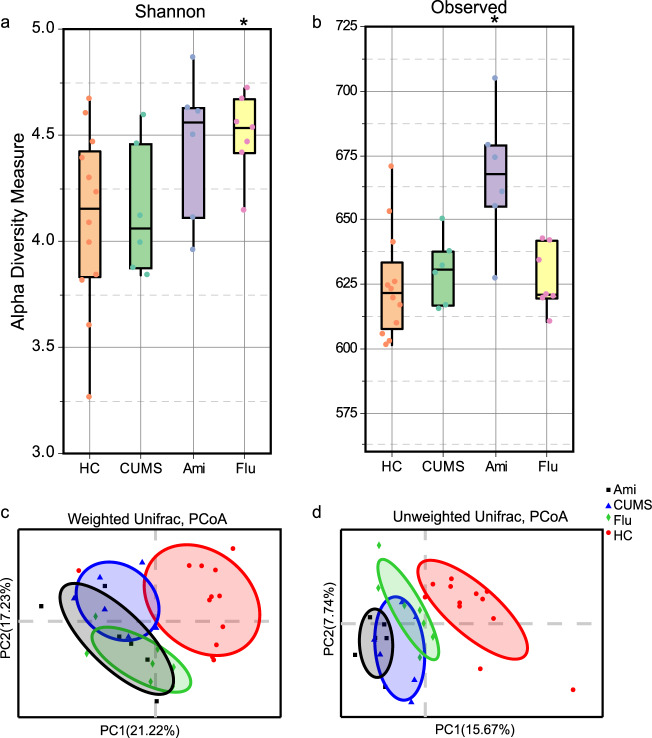
Measures of bacterial alpha- and beta-diversity. **a** ‘Shannon’ diversity considers both richness and evenness of OTUs within a sample. **b** ‘Observed’ diversity represents the number of OTUs (richness) present in each sample. **c** PCoA plots of bacterial beta-diversity based on weighted UniFrac distances at week 15. **d** PCoA plots of bacterial beta-diversity based on unweighted UniFrac distances at week 15. Kruskal–Wallis H followed by Tukey’s posthoc test performed to evaluate statistical significance. *P < 0.05 vs. CUMS.

All sample coverage (Good’s coverage) exceeded 99.50%, indicating that sequencing accuracy was reliable. Multiple rarefaction curves were measured using several metrics; i.e., Sobs, Chao 1, Simpson, and Shannon, which confirmed adequate sequence coverage for all samples (Supplementary Fig. 3a–d). To assess overall differences in microbial community structure among HC, CUMS, Ami and Flu groups, we calculated measures of alpha- and beta-diversity, for ecological diversity within a given sample, and between samples, respectively. Shannon Index, an alpha-diversity measure of both microbial richness and evenness, was enhanced following Ami treatment (*P* < 0.05*;* Fig. 2a). ‘Observed’ diversity, a measurement of OTU number, was increased after Flu treatment (*P* < 0.05; Fig. 2b). Although the alpha-diversity of CUMS rats was lower than that of HC rats, there was no statistically significant differences (*P* > 0.05).

**Fig. 3:**
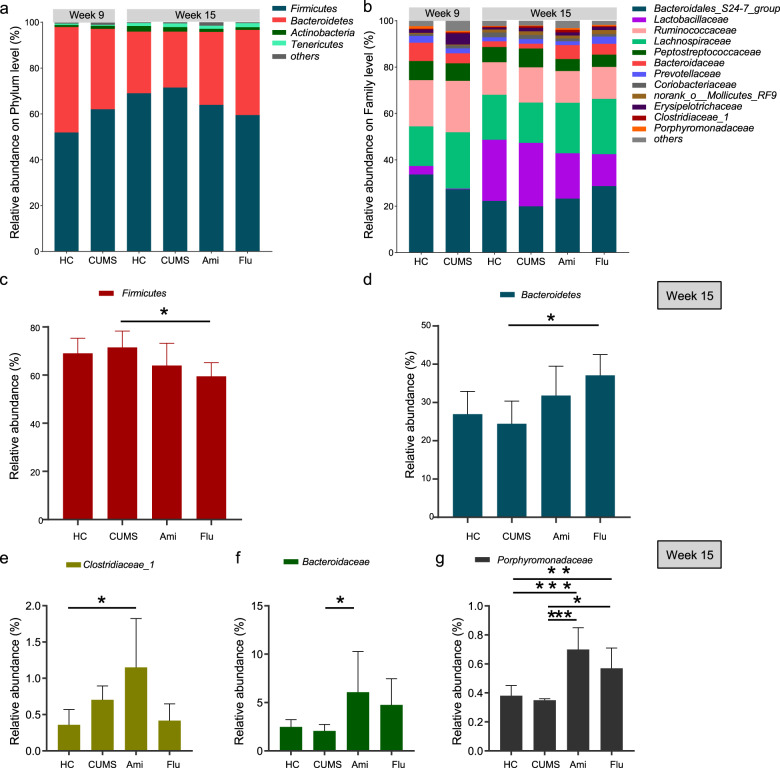
Faecal microbiota changes following antidepressant treatments at the phylum and family levels. Community bar-plot analysis shows the relative abundance of sequences at **a** the phylum and **b** family levels. OTUs comprising less than 1% at the phylum and family level of the total abundance are represented as ‘others’. Kruskal–Wallis H followed by Tukey’s posthoc tests statistics of the relative **c**, **d** bacterial phyla and **e**–**g** bacterial family abundances in HC, CUMS, Ami and Flu groups. Significant different are indicated: *P < 0.05, **P < 0.01, ***P < 0.001.

**Fig. 4:**
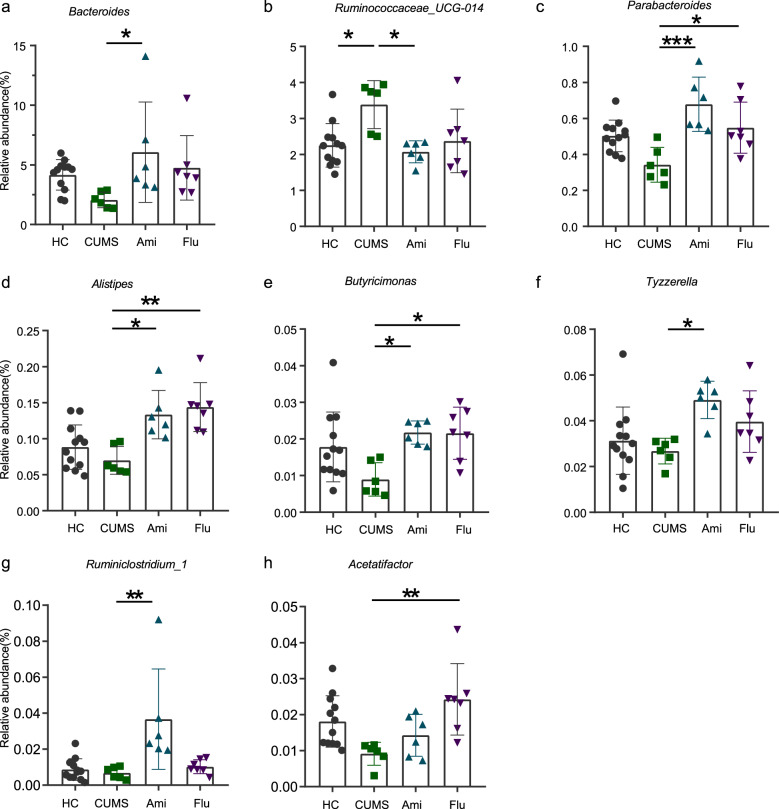
Faecal microbiota changes following antidepressants treatment at the genus level. Kruskal–Wallis H followed by Tukey’s posthoc tests statistics of the relative **a**–**i** bacterial genera abundance in HC, CUMS, Ami, and Flu groups. Significant different are indicated: *P < 0.05, **P < 0.01, ***P < 0.001.

**Fig. 5:**
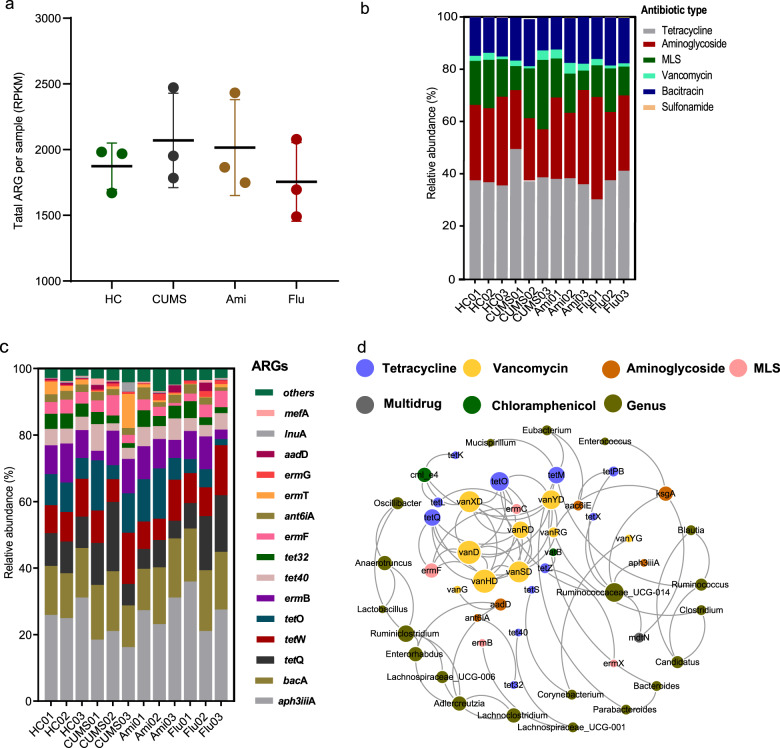
Antibiotic resistance gene analysis via metagenomics sequencing. **a** Scatter plot of the total ARG reads per kilobase per million mapped reads per sample (RPKM). **b** Relative ARG abundance per antibiotic type. **c** Relative abundance of the 15 most common ARGs. ARG abundance comprising less than 1% of the total abundance in all samples are represented as ‘others’. **d** Co-occurrence network analysis showing the correlation between resistance genes and bacterial taxa at the genus level. Only connections with a strong (Spearman’s *ρ* > 0.8) and significant (*P*-value < 0.01) correlation are presented in the network. The size of the nodes (circles) is proportional to the number of connections (the degree). MLS Macrolide–Lincosamide–Streptogramin resistance.

**Fig. 6:**
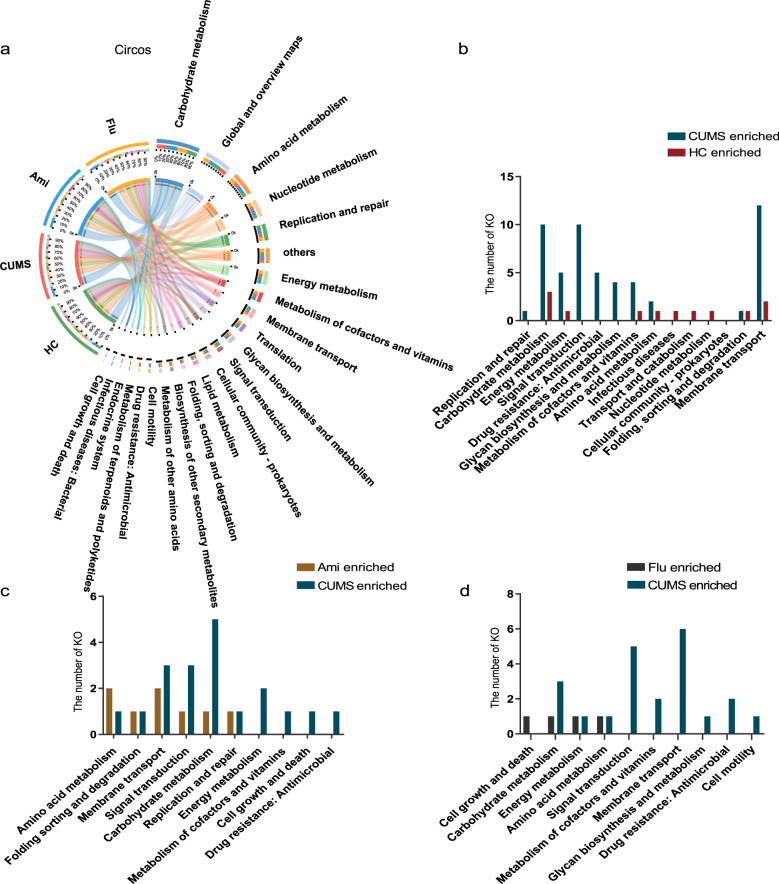
KEGG metabolic pathway analysis via metagenomics sequencing. **a** Distribution of KEGG metabolic pathways at level 2. **b** Comparison between the HC-enriched and CUMS-enriched KOs on level 2 of KEGG functional category. **c** Comparison between the CUMS-enriched and Ami-enriched KOs on level 2 of the KEGG functional category. **d** Comparison between the CUMS-enriched and Flu-enriched KOs on level 2 of the KEGG functional category. The abundance of KO in one group is significantly higher than that of other groups, indicating enrichment.

To determine whether overall microbial community structure differed among the four groups, we calculated the differences in beta-diversity using the weighted and unweighted UniFrac metric and visualised communities by PCoA (Fig. 2c, d). The PCoA results showed that the microbial communities in the CUMS group exhibited a distinct composition compared to that of HC rats, despite their access to similar food and water. However, the gut microbiota profiles of the antidepressant-treated rats demonstrated separation from those of CUMS rats, as assessed by the PERMANOVA test for clustering of weighted and unweighted UniFrac distances (*P* < 0.05; Supplementary Table 2). Notably, PCoA plots based on the metric indicated that the difference between the microbial communities of rats in the HC and CUMS groups at week 9 is greater than that at week 15 (Supplementary Fig. 4a, b). The natural maturation of gut microbiota during growth even in the control group HC (Fig. 3a, b) and potentially reduced susceptibility to the same types of stress after extended exposure in CUMS rats might contribute to the phenomenon. Also, the microbial community of the CUMS group at week 9 was far away from other groups, leading to the change of the relative distance among the remaining groups in the presentation. Thus, although using the same data, it appeared that CUMS and HC groups at week 15 were separated in the PCoA plots in Fig. 2c, d, but differed in Supplementary Fig. S4a, b. Regardless, even though CUMS and HC groups at week 15 were relatively close in the PCoA plots, the phylum, family, genus levels and functions of faecal microbiota were still distinct, as illustrated by the DNA sequence data as presented in Figs. 3–6.

### Oral antidepressants alter the relative abundance of faecal microbiota

At the phylum level, 16S rRNA gene sequencing data illustrated that the alterations of principal faecal microbiota between HC and CUMS rats were similar at weeks 9 and 15 (Fig. 3a). Moreover, from the week 15 results we observed that Ami and Flu treatments were the key categorical variable for faecal microbiota alterations. To further assess the structural response of faecal microbiota to antidepressant treatment, bacterial communities of all four groups at week 15 were compared. There were 16 different phyla identified across all faecal samples, of which only four had more than 1% overall relative abundance, namely, Firmicutes (65.96%), Bacteroidetes (30.10%), Actinobacteria (1.73%), Tenericutes (1.49%). ‘Others’ represents microbiota that constituted <1% of the abundance in all samples. Phylum level analysis showed a clear alteration of gut community associated with Ami and Flu characterised by a lower Firmicutes/Bacteroidetes ratio compared to CUMS rats, due to a reduced Firmicutes and increased Bacteroidetes. Moreover, the relative abundance in Firmicutes and Bacteroidetes reached a significant level between Flu and CUMS groups (*P* < 0.05; Fig. 3c, d and Supplementary Table 3).

At the family level, Bacteroidales_S24-7_group, Lactobacillaceae, Ruminococcaceae, Lachnospiraceae, Peptostreptococcaceae, Bacteroidaceae, Prevotellaceae, Coriobacteriaceae, norank_o__Mollicutes_RF9, Erysipelotrichaceae, Clostridiaceae_1, as well as other families, dominated in all four groups of rats, although changes in the relative abundance at the family level were observed following antidepressant treatment (Fig. 3b). Specifically, the sequences from Porphyromonadaceae were significantly increased in the Ami and Flu groups compared to those in CUMS and HC, while the abundance was similar between HC and CUMS. We also observed that antidepressants increased the abundance of Bacteroidaceae. However, only the Ami group exhibited significant differences as compared to CUMS (*P* < 0.05). Ami and Flu treatment were also associated with decreased taxon abundance, including family Lactobacillaceae and Peptostreptococcaceae, although the difference in relative abundance did not reach statistical significance (*P* > 0.05). Notably, the family Clostridiaceae_1 was only overrepresented in the Ami group and reached a significant level with respect to HC (*P* < 0.05; Fig. 3e–g and Supplementary Table 3).

At the genus level, we observed a significant effect elicited by antidepressant treatment with regard to increased *Bacteroides*, *Parabacteroides*, *Alistipes*, *Butyricimonas*, *Tyzzerella*, and *Acetatifactor* abundance and decreased *Ruminococcaceae_UCG-014* (Fig. 4a–h). Among these, *Bacteroides*, *Parabacteroides, Butyricimonas* and *Ruminococcaceae_UCG-014* reached levels similar to those of HC. In addition, compared to that of CUMS, only Flu treatment resulted in a higher abundance of *Acetatifactor*. In the antidepressant groups, the increase in Bacteroidetes corresponded to a higher abundance of *Bacteroides* and *Parabacteroides* along with other bacterial components present in low abundance. In comparison, the decrease in Firmicutes was primarily due to the relatively lower abundance of *Ruminococcaceae_UCG-014*.

To further investigate whether the alterations in faecal microbiota were associated with anxiety-like and depressive-like symptoms, Spearman rank correlation analysis was performed for the differential taxa and behaviour indices between the CUMS and antidepressant-treated rats. Following Ami treatment, we observed that altered faecal microbiota in Porphyromonadaceae, *Ruminiclostridium_1*, *Parabacteroides*, *Alistipes*, and *Tyzzerella* positively correlated with the number of rearing and crossing counts in the OFT (Supplementary Fig. 5a). Meanwhile, in the Flu-treated animals, the *Acetatifactor* genus negatively correlated with the LDT indices and positively correlated with SPT and OFT results (Supplementary Fig. 5b).

To further validate the potential impact elicited by antidepressants on the microbial composition mentioned above, we also used the shotgun sequencing data for microbiota profile assessment. The abundances of five species were significantly higher and 21 species were significantly lower in the CUMS rats than those in the HC rats (Supplementary Fig. 6a). Notably, we observed four *Bacteroides* species were enriched in the HC group compared to the CUMS group, which was consistent with the 16S sequencing analysis results. Similar results were observed in the antidepressants treatment group compared with the CUMS group. Specifically, eight *Bacteroides*, *Parabacteroides* and *Butyricimonas* species were significantly increased in the Ami group, while three *Alistipes* species were enriched in the Flu group (Supplementary Fig. 6b, c). Hence, although Ami and Flu are both antidepressants, they exhibited different impacts on the faecal microbial composition. Specifically, compared with Flu, Ami treatment significantly enhanced the abundance of several *Bacteroides* and *Butyricimonas* species (Supplementary Fig. 6d). Although Flu also increased the abundance of both genera in CUMS rats according to metagenomic sequencing data, the effect of Ami may be more potent, particularly regarding the altered abundance of *Bacteroides*.

### Influence of antidepressants on faecal microbiota antibiotic resistance genes

Analysis of the ARG profiles of faecal samples in the HC, CUMS, Ami, and Flu groups revealed that the total abundance of ARGs was similar among the four groups, with no significant differences observed (Fig. 5a). As several different ARGs might encode resistance to the same type of antibiotic, the relative abundance of ARGs was aggregated to the corresponding antibiotic type for each sample. Results showed that for the four groups, ARGs encoding resistance towards macrolide–lincosamide–streptogramin (MLS), tetracycline, aminoglycoside and bacitracin were the most abundant (Fig. 5b). Meanwhile, faecal samples from both antidepressant treatment groups exhibited high relative proportions of the *aph3iii*A aminoglycoside resistance gene, whereas *erm*T among MLS resistance genes was more abundant in CUMS. Additionally, the *tet*O tetracycline resistance gene had low relative proportion in the Flu-treated rats (Fig. 5c, Supplementary Table 4). The category ‘Others’ represents ARGs that constituted <1% abundance in all samples. The overall patterns of ARGs were revealed by PCoA. Notably, the four groups could not be distinguished using PCoA, indicating that the differences among the groups were insignificant (Supplementary Fig. 7). Specifically, among 84 ARGs identified, 60 were shared in all samples.

To characterise the difference of ARGs between groups and to identify potential indicator ARGs, LDA Effect Size (LEfSe) algorithm module was used to couple statistical significance with biological consistency and effect size estimation. We observed that the abundance of indicator ARGs in antidepressant treatment groups were significantly different from that in the CUMS group (Supplementary Fig. 8a–c). The indicator ARGs found in Ami (*aph3iii*A, *tet*M, *ls*A, *catb*2, *bcR*, and *emr*D) comprised genes conferring resistance to aminoglycoside, tetracycline, MLS, and multidrug. In turn, the Flu group harboured nine indicator ARGs conferring resistance to multidrug (*mdt*K, *mdt*P, *mdt*H, *mdt*G, and *acr*A), aminoglycoside (*aph3iii*A), tetracyclines (*tet*M), and chloramphenicol (*cml_e*8). To assess the relationship between bacterial communities and ARG number and abundance, we analysed the relationship between microbial OTUs and ARG files of the faecal samples using the Mantel test. A significant correlation between the ARG abundance data and bacterial 16S rRNA gene OTU data of the gut microbiota was observed (*r* = 0.346, *P* = 0.01).

Moreover, ARG variation associated with the efflux pumps was analysed. Our results indicate that the ARGs for efflux proteins primarily conferred resistance to MLS, multiple drugs, and bacitracin, while others conferred resistance to aminoglycoside, quinolone and tetracycline (Supplementary Fig. 9 a–d). Compared to the HC group, the abundance of certain ARGs conferring resistance to MLS efflux pump (*ole*B, *lsa*C) was decreased, while *ade*H conferring resistance to multidrug efflux pump was enhanced in the CUMS group. Consistent with the HC group results, we observed increased abundance of *lsa*C conferring resistance to MLS efflux pump in Ami compared with CUMS rats. However, genes for efflux pumps had no apparent variation in the Flu group. This may have been caused by the small sample size or, the ARGs including multidrug efflux pumps may actually happen to be associated with bacterial subpopulation vary in their intrinsic sensitivity to the two antidepressants (Supplementary Fig. 10).

Co-occurrence patterns between ARGs and bacterial assemblages were explored based on strong (*ρ* > 0.8) and significant (*P* < 0.01) correlations (Fig. 5d). The analysed topological network properties are summarised in Supplementary Table 5. The detailed co-occurrence between ARGs and microbial taxa are summarised in Supplementary Table 6. A previous study hypothesised that non-random co-occurrence patterns can be used as potential host information for ARGs when their abundance and co-existing microbial taxa are significantly similar between the different samples^50^. As shown in Fig. 5d and in Supplementary Table 6, the co-occurrence analysis results indicated that 12 bacterial genera may serve as the potential hosts for the 20 AGRs. Among these 12 genera, *Ruminococcaceae*_UCG-014 carried more diverse ARGs than the other genera, with connections observed with aminoglycoside resistance genes (*aph3iii*A and *ksg*A), multidrug resistance gene (*mdt*N), and tetracycline resistance genes (*tet*O, *tet*PB, *tet*X). Additionally, the *Parabacteroides* and *Bacteroides* genera that were altered in the faecal microbiota analysis could be hosts of *tet*M genes, which encode tetracycline resistance.

### Effects of antidepressants on faecal microbiota KEGG metabolic pathways

To investigate the effects of Ami and Flu on the function of faecal microbiota, the data obtained from metagenomic analysis were annotated against the KEGG database. The highest abundance functional categories were associated with metabolism, which primarily consisted of carbohydrate, amino acid, energy, and nucleotide metabolism (Fig. 6a). Analysis of 6,101 KEGG orthologues (KOs) revealed that the relative abundance of 124 KOs notably differed between HC and CUMS groups. The 102 CUMS-enriched KOs were typically involved in the KEGG categories of ‘membrane transport’, ‘carbohydrate metabolism’, and ‘signal transduction’ (Fig. 6b, Supplementary Table 7). Ami and Flu treatment caused a difference in 54 and 70 KOs, respectively, compared to those in the CUMS group. Notably, following Ami intervention for 6 weeks, an apparent reduction was observed in the abundance in KOs related to ‘carbohydrate metabolism’, ‘signal transduction’, and ‘membrane transport’ (Fig. 6c and Supplementary Table 7). These results are consistent with the findings in Flu rats (Fig. 6d and Supplementary Table 7).

Additionally, using LEfSe analysis, we observed that pathways associated with translation (ribosome), drug resistance: antimicrobial (cationic antimicrobial peptide (CAMP) resistance), amino acid metabolism (glycine, serine, and threonine metabolism), and metabolism of terpenoids and polyketides (terpenoid backbone biosynthesis) were decreased in Ami rats (Supplementary Fig. 11b), in agreement with the decreased functions in the HC group (Supplementary Fig. 11a). In contrast, pathways related to replication and repair (nucleotide excision repair) and immune system (NOD-like receptor signalling pathway) were enriched in the Ami group, consistent with the enriched functions in the HC group. Analysis of the Flu-treated CUMS rats revealed enrichment of only the ‘glutathione metabolism’ pathway, which was not observed in the HC group (Supplementary Fig. 11c).

## Discussion

Drugs can affect the function and composition of gut microbiome^53^. Most studies on antidepressants have focused on the effects associated with clinical symptoms of patients with depressive disorder, such as disturbances in sleep, appetite, and desire, as well as constipation, loss of the ability to experience pleasure in work or with friends, crying, suicidal thoughts, and slowing of speech and action^2^. However, very limited studies have evaluated the effects on intestinal microbiota^25,27^, and none have evaluated the influence of Flu or Ami on the structure and function of intestinal microorganisms under conditions of depressed hosts. In this study, Ami and Flu were shown to reduce the depressive symptoms of CUMS rats, including increasing the sucrose preference ratio, improving activity and exploration, and decreasing the time spent in the dark zone, suggesting the important roles of antidepressants in mediating the anxiety- and depression-like phenotype in CUMS rats. Data of 16S rRNA gene sequencing of faecal samples illustrated increased alpha-diversity in the bacterial communities of the antidepressant treatment groups compared to that in the CUMS rats. While bacterial diversity is believed to be important in host health, how the diversity of the microbiome affecting health remains largely unanswered^54^. More importantly, data on the potential association between depression and alpha-diversity are so far inconsistent. For example, Jiang et al.^55^ and Kelly et al.^17^ reported an increase in faecal bacterial alpha-diversity in depressed patients, whereas both Naseribafrouei et al.^15^ and Zheng et al.^19^ did not observe any significant alteration in human with depression. In the present study, beta-diversity analysis using weighted and unweighted UniFrac metrics revealed that the microbial communities in the CUMS group had a distinct composition compared with those in the HC rats, despite their access to similar food and water, supporting the potential impact of chronic stressors on the profile of gut microbiota. Then, the gut microbiota profiles of the Ami and Flu rats exhibited a separation from those of CUMS rats, suggesting that the antidepressant treatment condition constituted a critical factor accounting for the change in microbial structure. This finding is consistent with previous research demonstrating that antidepressant medication is an important source of inter-study variation regarding the gut microbiota composition of individuals^56^. Moreover, we identified that lower abundance of the phylum Firmicutes and higher abundance of phylum Bacteroidetes were associated with the drug-treatment effects of Ami and Flu, with that of the Flu group reaching a significant level. In comparison, data obtained from human studies and animal models have revealed that the Firmicutes/Bacteroidetes proportion is decreased in lean compared to obese subjects and tends to increase with weight gain^57,58^. Additionally, several neurological diseases and inflammatory conditions have been related to an increase in the Firmicutes/Bacteroidetes ratio, such as autism spectrum disorders^59–61^and inflammatory bowel diseases^62^. Therefore, a reduced ratio of Firmicutes/Bacteroidetes appears to be associated with an improvement in neurological condition.

At the genus level, we discovered that the relative abundances of *Bacteroides*, *Parabacteroides*, *Butyricimonas*, *Acetatifactor*, and *Tyzzerella* have significantly increased in the faecal microbiota of Ami and Flu rats as compared to those in the CUMS rats, whereas the relative abundance of the genus *Ruminococcaceae_UCG-014* was significantly reduced in Ami and Flu rats. Additionally, we found that the abundance of *Bacteroides* was decreased in CUMS rats compared to that in HC rats. Notably, previous studies indicated that a decreased abundance of *Bacteroides* is associated with several diseases, such as obesity and diabetes^63,64^, while depression and metabolic disease comorbidity is also common^65^. Our data are consistent with results from previous human studies that *Bacteroides* expression was significantly decreased in patients with active major depressive disorder^55^. In addition, members of *Bacteroides* and *Parabacteroides* have been reported to actively express pathways producing GABA, a major inhibitory neurotransmitter with a prominent role in the brain control of stress^66^. Currently used antidepressants, which are designed to augment monoaminergic transmission, all ultimately serve to enhance GABAergic transmission^67^. Moreover, we observed decreased levels of *Butyricimonas* in CUMS rats. Notably, the genus *Butyricimonas* are butyrate producers with anti-inflammatory properties^68^. Butyrate is related to the reduction of inflammation and helps maintain a healthy gut^69^. In the present study, following antidepressant treatment, the levels of *Butyricimonas* in the Ami and Flu groups reached a similar level to that in HC. Therefore, Ami and Flu antidepressant treatments likely impacted gut microbiota directly, or indirectly through host factors, including those of significance to brain health, as mentioned above.

However, Ami and Flu treatments may also affect microbial subpopulation with negative health impact. In particular, the abundance of Porphyromonadaceae family members and genus *Alistipes* were found increased in Ami and Flu rats, whereas the abundance was similar in HC and CUMS animals. Previous studies have revealed that lower Porphyromonadaceae abundance served as a specific bacterial signature of inflammation suppression^70^ in fat mass- and obesity-associated gene deficiency mice and that some species in *Porphyromonadaceae_unclassified* were related to brain inflammation^71,72^. Furthermore, enrichment of *Alistipes* has been reported in patients with gastrointestinal complications^73^, chronic fatigue syndrome^74^, and depression^15^. In Il10^–/–^ mice models, *Alistipes* have been shown to induce colitis and tumours^75^. Notably, *Alistipes* species are indole-positive and may, therefore, influence tryptophan availability^76^. As tryptophan is also the precursor of serotonin, increased abundance of *Alistipes* might disrupt the balance in the intestinal serotonergic system^55^. Consistent with this conjecture, Saulnier et al.^77^ found that higher levels of *Alistipes* were associated with a greater frequency of abdominal pain in patients with irritable bowel syndrome and speculated that *Alistipes* is associated with inflammation, as was observed for the family Porphyromonadaceae. Although the link between antidepressants and potentially harmful bacteria has not been explored, studies have indicated potentially serious adverse events associated with the use of antidepressant drugs, such as gastrointestinal symptoms, metabolic abnormalities and cardiovascular disturbances^33,78^. Thus, further research is needed to clarify the role(s) of this family and genus in the outcomes, including adverse effects of antidepressant treatment.

Recently, the correlation between non-antibiotic drugs and bacterial antibiotic resistance has attracted considerable attention^34^. For example, an in vitro study reported that Flu can induce resistance to multiple antibiotics in *Escherichia coli* via reactive oxygen species-mediated mutagenesis^79^. However, little is known regarding the effects of antidepressants on ARGs in vivo. In this study, we used linear discriminant analysis as a tool to discover potential indicator ARGs. The results suggest that altered ARG abundance is associated with antidepressant treatment. Particularly, this could lead to the enrichment of certain bacteria with multidrug resistance mechanisms to affect their future susceptibility to Flu, or it could simply be a coincidence that bacteria with less susceptibility to Flu carry these genes and were, therefore, enriched when sensitive ones were inhibited. Furthermore, in the present study, network analysis revealed a strong correlation between the bacterial community and ARG profiles in the faecal samples. Therefore, the antimicrobial effects of antidepressants should be rationally considered, while the importance of monitoring the profiles of microbes and ARGs in patients with depression should not be underestimated.

Additionally, our metagenomics data suggested systemic metabolic alterations following antidepressant treatment. Since the metabolic functions of microbiota associated with depression remain largely unknown, results from this study opened the door for future studies. Specifically, our data illustrated an elevation in the gut microbial function of membrane transport, carbohydrate metabolism, and signal transduction in CUMS rats, whereas Ami and Flu treatment led to a decrease in these functions. The enhanced carbohydrate metabolic pathways in CUMS compared with control rats potentially suggest that a higher energy demand exists in depressed rats if such pathways are not just simply associated with microbial subpopulations less susceptible to host response(s) triggered by stressors. Moreover, a previous study has also reported that the KEGG category of membrane transport is enriched in patients with inflammatory bowel disease (IBD)^80^, while patients of the depressive disorder often suffer from IBD^81,82^. Overall, these data support the notion that administration of Ami and Flu potentially have additional health implications beyond anti-depression, due to its impact on gut microbiota metabolic functions.

It is important to recognise that gut microbiota functional analysis is based on shotgun sequencing data of very short DNA fragments from the faecal microbiota DNA extracts, and further uses mathematic calculations to predict the metabolic pathways. Therefore, the data should only be considered as indicative reference rather than solid evidence. The availability of an enriched database of whole-genome sequences for related gut bacteria and full annotation of their metabolic pathways and related genes will enable proper interpretation of the gut microbiota functions.

In summary, results from this study illustrated that CUMS-induced gut microbiota disruption in rats, with featured changes in community diversity, taxon abundance, and function profiles, along with in depressive symptoms. Administration of Ami and Flu reversed part of the gut microbiota profile and functions, in line with the antidepressant effects. Particularly, both antidepressant treatments led to an altered abundance of Porphyromonadaceae, *Bacteroides*, *Parabacteroides,* and *Alistipes*. Ami and Flu treatments further modulated the abundance of several ARGs and potentially reduced carbohydrate metabolism in CUMS rats. Results from the study laid the foundation for further investigations to characterise the mechanisms of Ami and Flu as antidepressants, to identify the metabolites mediating the host responses, and to validate the causative relationship between gut microbiota dysbiosis and depression by demonstrating the function of the specific bacteria in hosts. Furthermore, so far antidepressant treatment-induced remission of depression remains an unsolved puzzle in disease therapy. Understanding the interaction between antidepressants and gut microbiota may open the door for innovative interpretation and potentially targeted intervention

## Supplementary information

Supplementary figure legends

Supplementary Figure 1

Supplementary Figure 2

Supplementary Figure 3

Supplementary Figure 4

Supplementary Figure 5

Supplementary Figure 6

Supplementary Figure 7

Supplementary Figure 8

Supplementary Figure 9

Supplementary Figure 10

Supplementary Figure 11

Supplementary Table 1

Supplementary Table 2

Supplementary Table 3

Supplementary Table 4

Supplementary Table 5

Supplementary Table 6

Supplementary Table 7

## Data Availability

The datasets generated during the current study are available in the Sequence Read Archive (SRA) database repository accession numbers PRJNA609527 and PRJNA663847.
