# Supplementary material for: Antidepressants fluoxetine and amitriptyline induce alterations in intestinal microbiota and gut microbiome function in rats exposed to chronic unpredictable mild stress
Source: Transl Psychiatry. 2021 Feb 18;11:131. doi: 10.1038/s41398-021-01254-5 (PMC7892574; doi:10.1038/s41398-021-01254-5)
Supplement: Supplementary file 1 — Supplementary figure legends [file 41398_2021_1254_MOESM1_ESM.docx]

**Suppl Fig. 1** Experimental schedule of the present study (15 weeks).

**Suppl Fig. 2** Hierarchical clustering tree analysis was performed at operational taxonomic unit (OTU) level for SEN and RES group. (SEN: rats are sensitive to CUMS; RES: rats are resistant to CUMS).

**Suppl Fig. 3** Rarefaction curves of sequences. Multiple rarefaction curves of sequences obtained from all samples for (**a**) Shannon, (**b**) Chao, (**c**) Simpson, and (**d**) Sob index.

**Suppl Fig. 4** PCoA of bacterial beta diversity based on the (**a**) weighted and (**b**) unweighted UniFrac distances according to different time periods. Healthy control rats (HC) at week 9, chronic unpredictable mild stress rats (CUMS) at week 9, healthy control rats (HC) at week 15 and chronic unpredictable mild stress rats (CUMS) at week 15 are colored in red, green, blue and yellow, respectively.

**Suppl Fig. 5** Spearman correlations between the altered fecal microbiota and behavior indices. **a** Spearman’s rank correlation coefficient of behavior indices and fecal microbiota that differed significantly between the CUMS rats and the Ami rats. **b** Spearman’s rank correlation coefficient of behavior indices and fecal microbiota that differed significantly between the CUMS rats and the Flu rats. Sucrose preference test (SPT); open filed test (OPT); light/dark test (LDT). Values of significance test are indicated: **P* < 0.05, ***P* < 0.01.

**Suppl Fig. 6** Changes in microbial species composition were analyzed via metagenomics sequencing. **a** Altered composition in the gut microbial species between HC and CUMS groups. **b** Altered composition in the gut microbial species between CUMS and HC groups. **c** Altered composition in the gut microbial species between CUMS and Flu groups. **d** Altered composition in the gut microbial species between Ami and Flu groups.

**Suppl Fig. 7** The PCoA analysis of the gut bacterium antibiotic resistance genes (ARGs). Principal-coordinate analysis (PCoA) analysis displaying variation of antibiotic resistance genes (ARGs) among four groups (n=3) based on the Bray-Curtis distances.

**Suppl Fig. 8** Antibiotic resistance genes (ARGs) divergence between gut microbiota of HC, Ami, Flu and CUMS groups. **a** Key ARGs for discriminating the fecal microbiomes of the CUMS and HC group. **b** Key ARGs for discriminating the fecal microbiomes of the CUMS and Ami rats. **c** Key ARGs for discriminating the fecal microbiomes of the CUMS and Flu rats. The threshold value greater than 2 was used as the cutoff value for statistical significance based on a *P*-value of 0.05 in the LDA analysis.

**Suppl Fig. 9** ARGs for efflux pumps were classified based on antibiotics to which they confer resistance. MLS: Macrolide-Lincosamide-Streptogramin.

**Suppl Fig. 10** ARGs-level heat map. Relative abundance of the ARGs in HC, CUMS, Ami and Flu groups. Colors represent log (ln) transformed relative abundances. MLS: Macrolide-Lincosamide-Streptogramin.

**Suppl Fig. 11** KEGG metabolic pathways divergence between gut microbiota of HC, Ami, Flu and CUMS groups. **a** Key KEGG pathways for discriminating the fecal microbiomes of the CUMS and HC group. **b** Key KEGG pathways for discriminating the fecal microbiomes of the CUMS and Ami rats. **c** Key KEGG pathways for discriminating the fecal microbiomes of the CUMS and Flu rats. The threshold value greater than 2 was used as the cutoff value for statistical significance based on a *P*-value of 0.05 in the LDA analysis.
