# Supplementary material for: Antidepressants fluoxetine and amitriptyline induce alterations in intestinal microbiota and gut microbiome function in rats exposed to chronic unpredictable mild stress
Source: Transl Psychiatry. 2021 Feb 18;11:131. doi: 10.1038/s41398-021-01254-5 (PMC7892574; doi:10.1038/s41398-021-01254-5)
Supplement: Supplementary file 2 — Supplementary Figure 1 [file 41398_2021_1254_MOESM2_ESM.pdf]

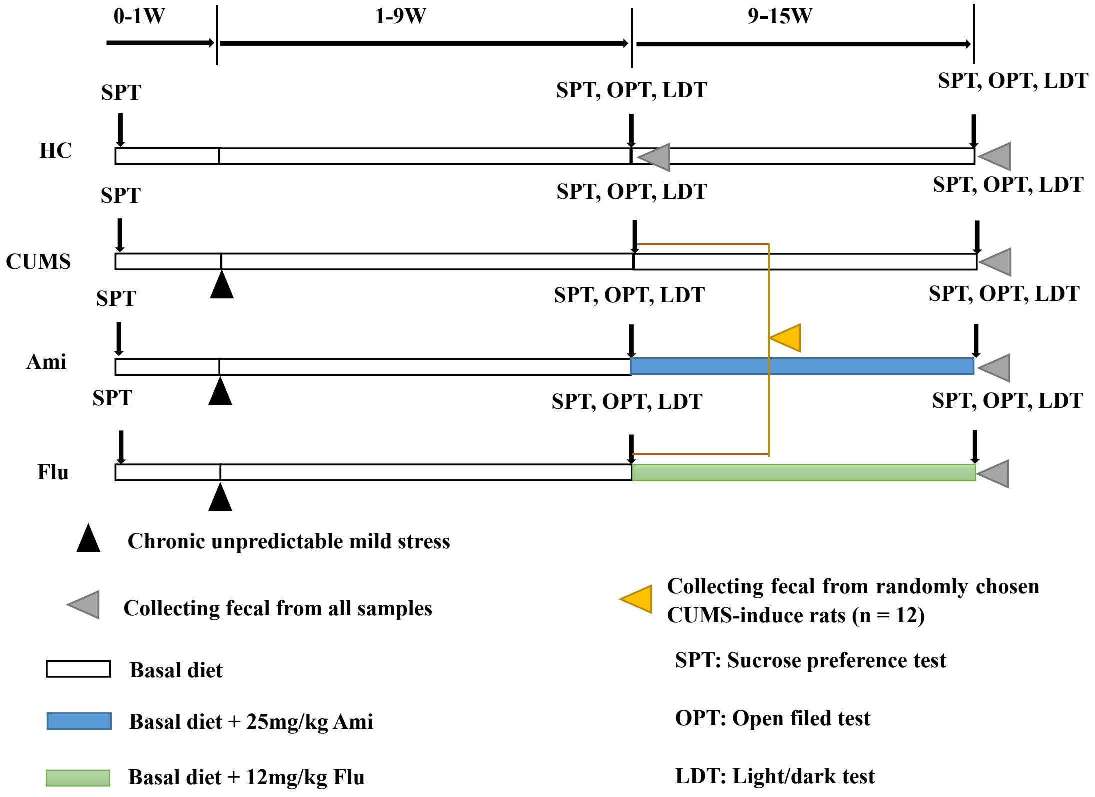
0-1W
1-9W
9-15W
SPT
SPT, OPT, LDT
SPT, OPT, LDT
HC
SPT
SPT, OPT, LDT
SPT, OPT, LDT
CUMS
SPT
SPT, OPT, LDT
SPT, OPT, LDT
Ami
SPT
SPT, OPT, LDT
SPT, OPT, LDT
Flu
Chronic unpredictable mild stress
Collecting fecal from all samples
Collecting fecal from randomly chosen CUMS-induce rats (n = 12)
Basal diet
Basal diet + 25mg/kg Ami
Basal diet + 12mg/kg Flu
SPT: Sucrose preference test
OPT: Open filed test
LDT: Light/dark test
