# Supplementary figures and images for: Antidepressants fluoxetine and amitriptyline induce alterations in intestinal microbiota and gut microbiome function in rats exposed to chronic unpredictable mild stress
Source: Transl Psychiatry. 2021 Feb 18;11:131. doi: 10.1038/s41398-021-01254-5 (PMC7892574; doi:10.1038/s41398-021-01254-5)

Hierarchical clustering tree on OTU level

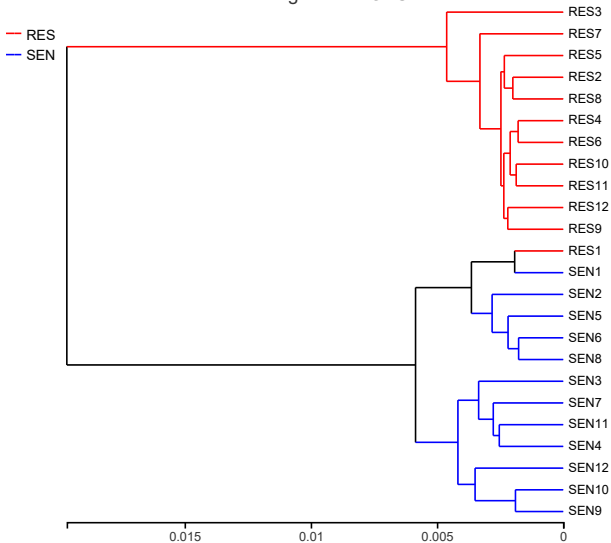

Supplement: Supplementary file 3 — Supplementary Figure 2 [file 41398_2021_1254_MOESM3_ESM.pdf]

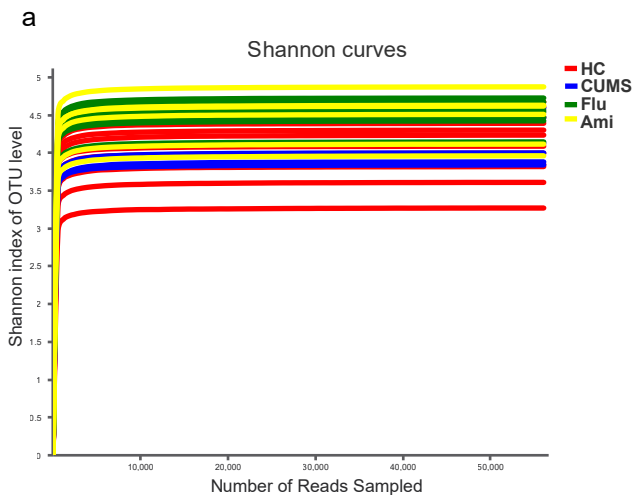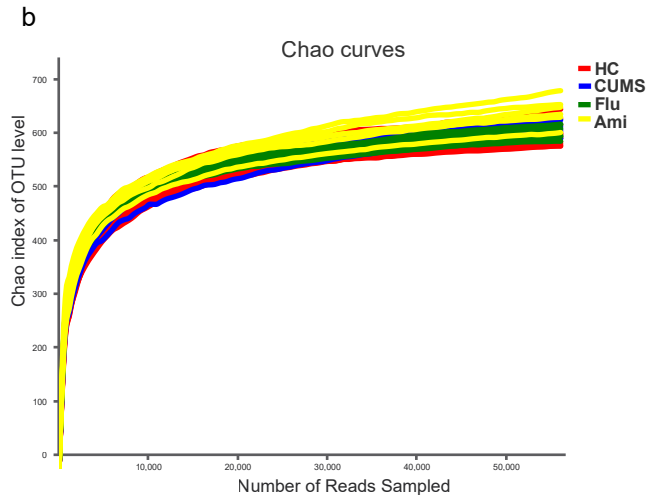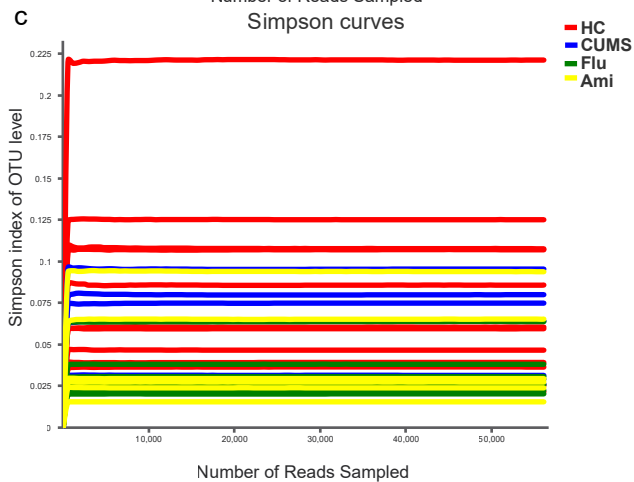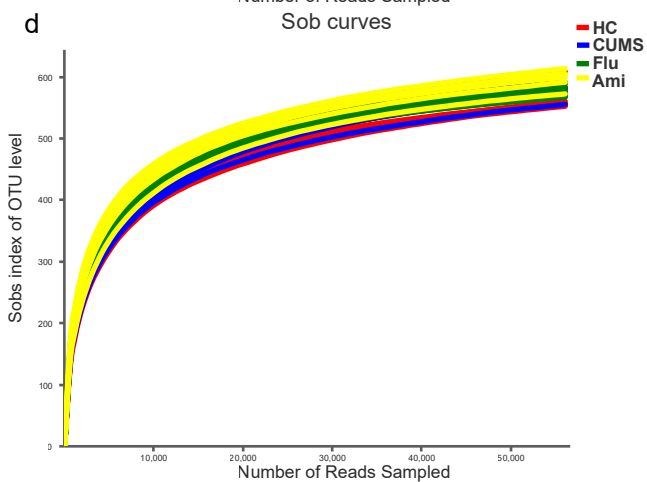

Supplement: Supplementary file 4 — Supplementary Figure 3 [file 41398_2021_1254_MOESM4_ESM.pdf]

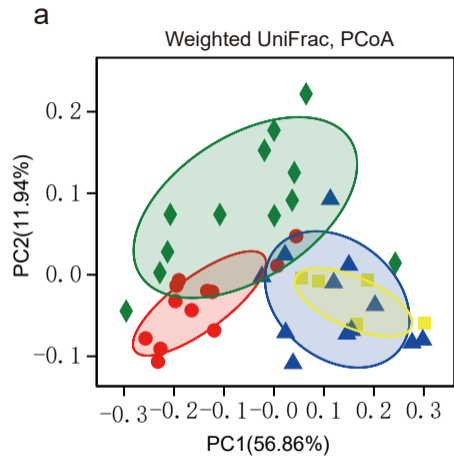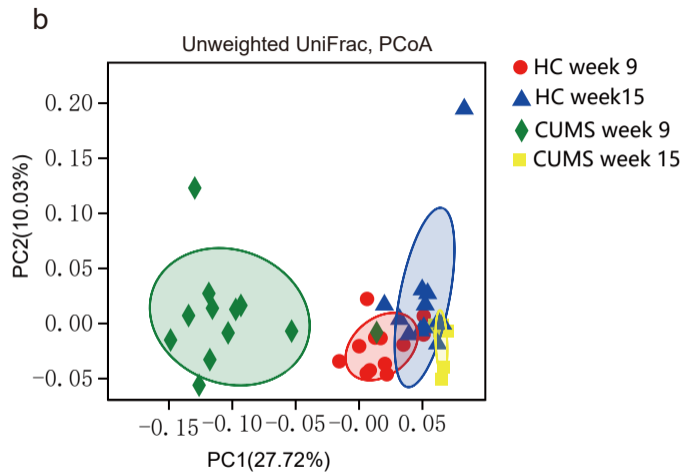

Supplement: Supplementary file 5 — Supplementary Figure 4 [file 41398_2021_1254_MOESM5_ESM.pdf]

a

LEfSe Bar

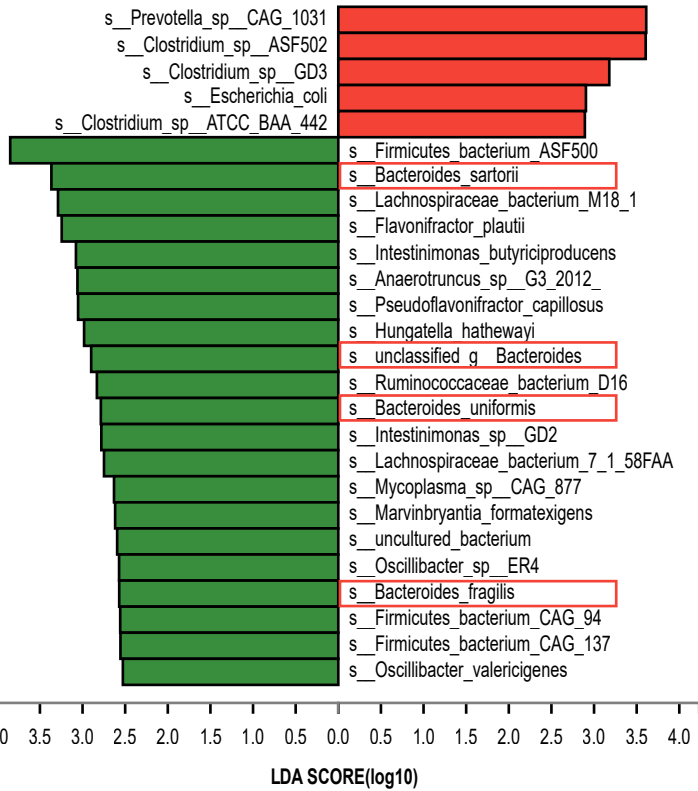

b

LEfSe Bar

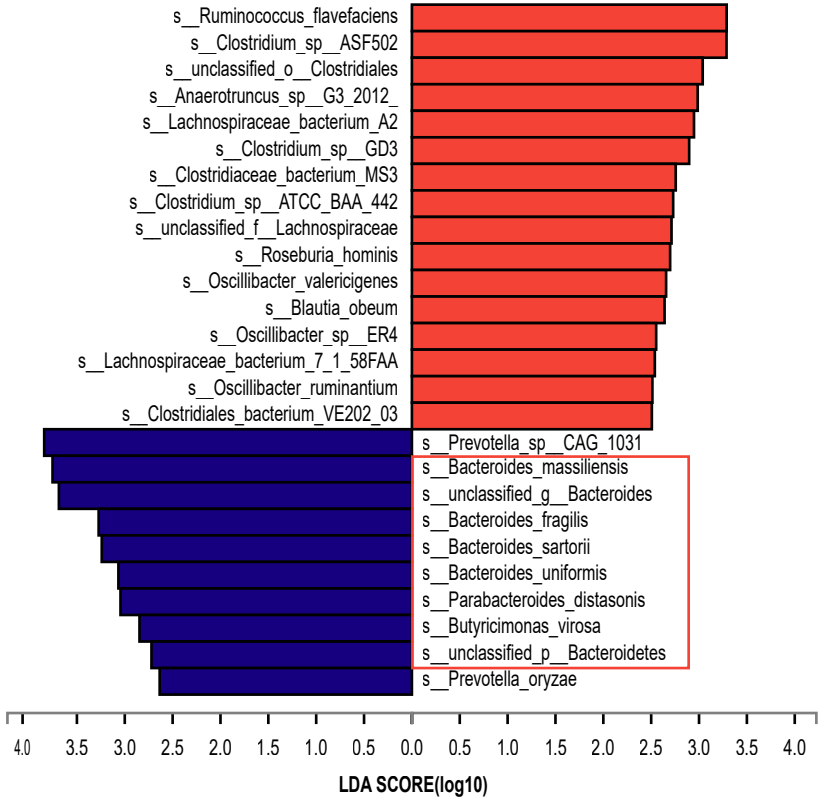

c

LEfSe Bar

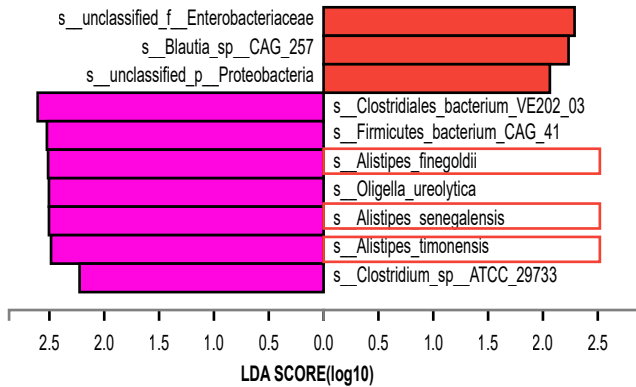

d

LEfSe Bar

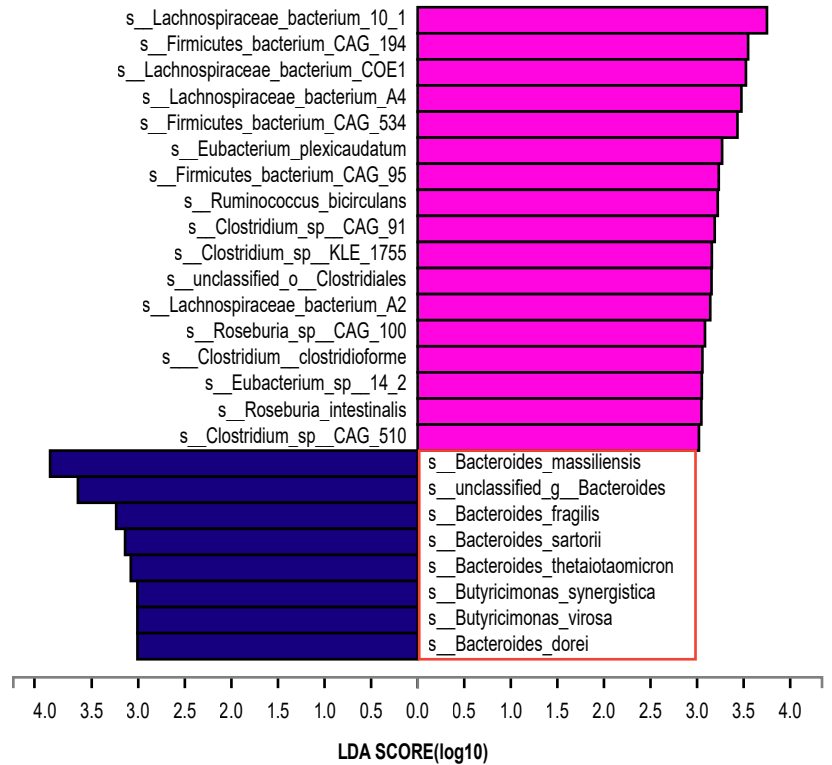

Supplement: Supplementary file 7 — Supplementary Figure 6 [file 41398_2021_1254_MOESM7_ESM.pdf]

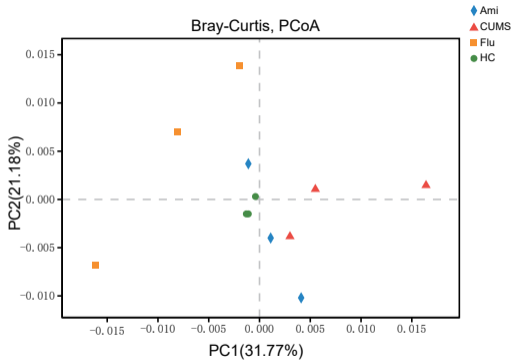

Supplement: Supplementary file 8 — Supplementary Figure 7 [file 41398_2021_1254_MOESM8_ESM.pdf]

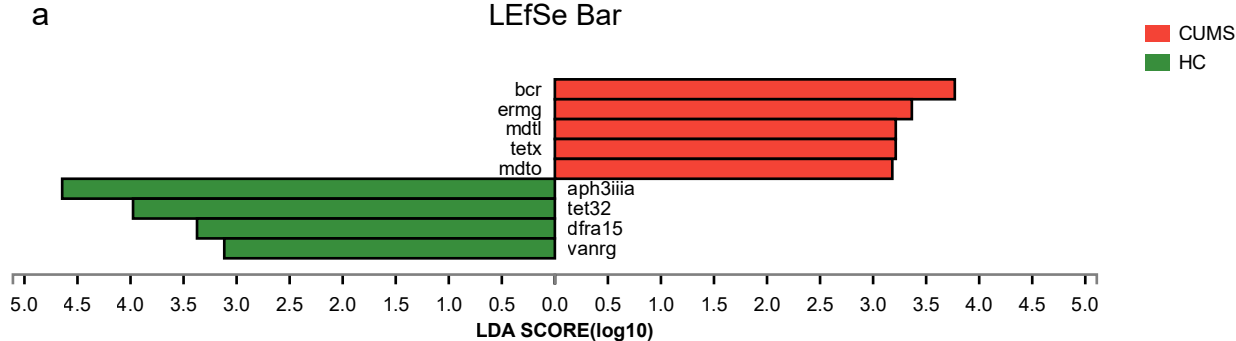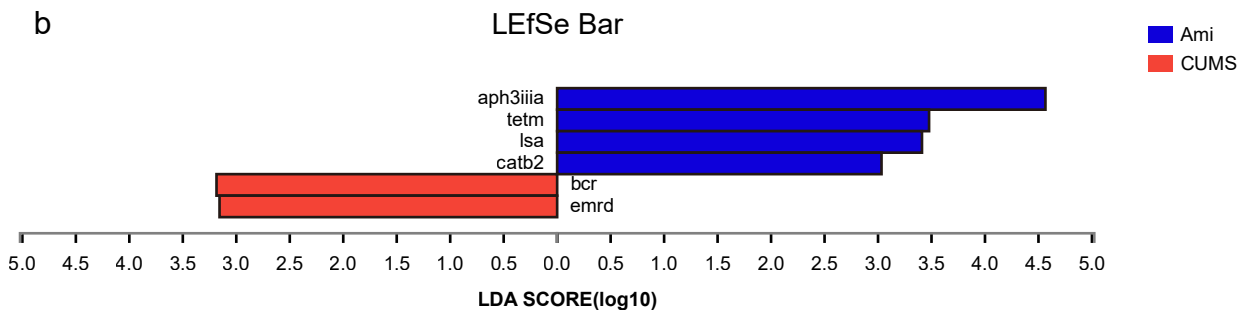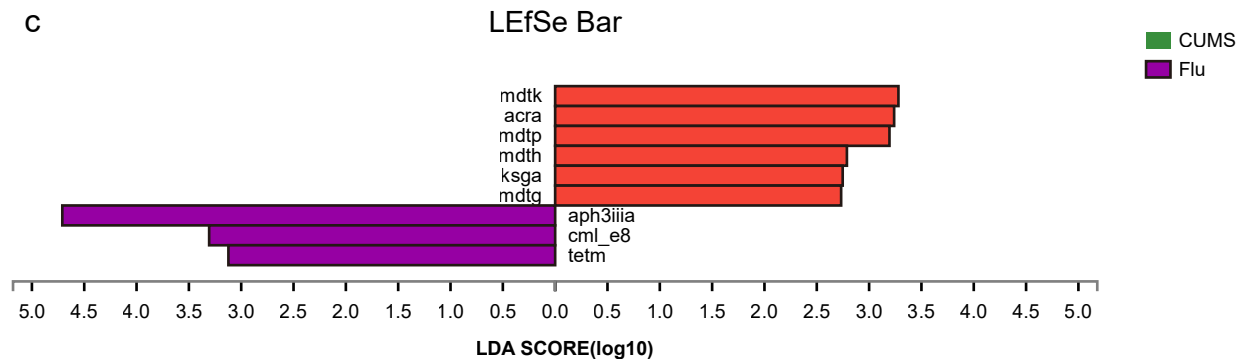

Supplement: Supplementary file 9 — Supplementary Figure 8 [file 41398_2021_1254_MOESM9_ESM.pdf]

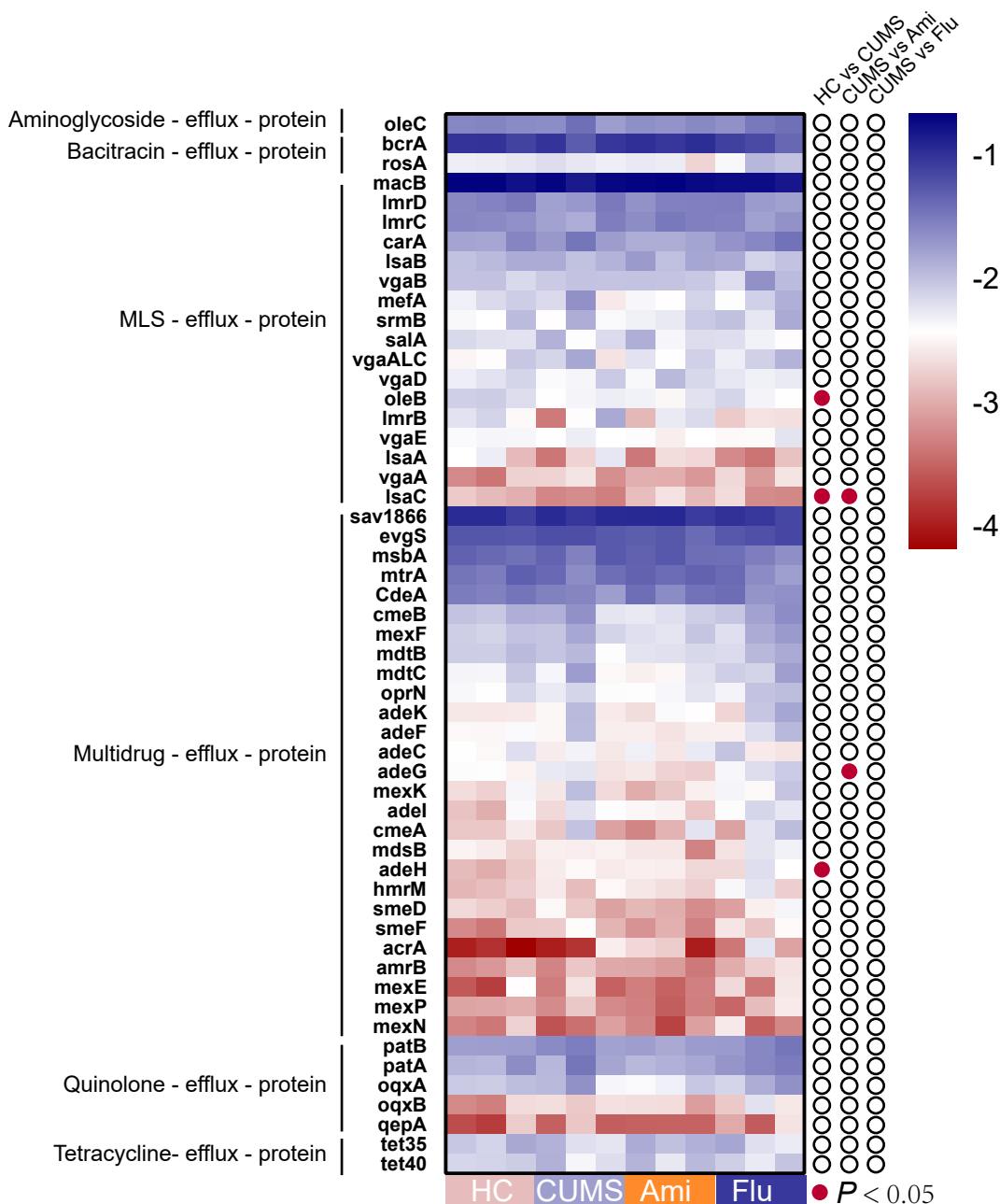

Supplement: Supplementary file 11 — Supplementary Figure 10 [file 41398_2021_1254_MOESM11_ESM.pdf]

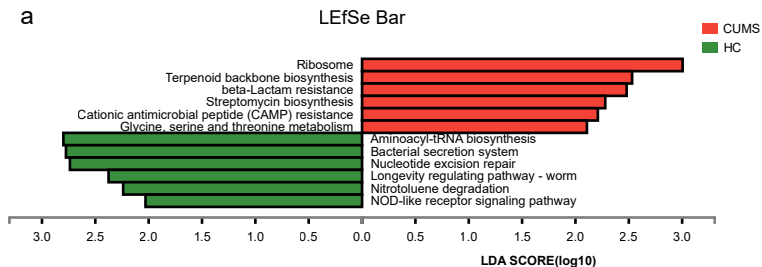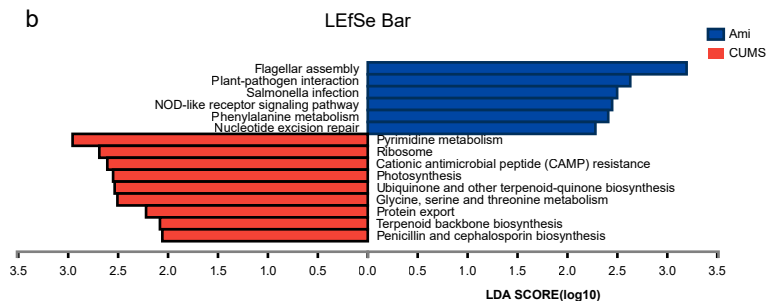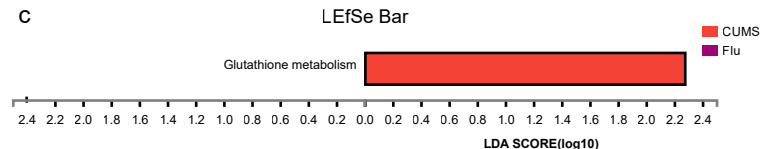

Supplement: Supplementary file 12 — Supplementary Figure 11 [file 41398_2021_1254_MOESM12_ESM.pdf]
