# Supplementary material for: Antidepressants fluoxetine and amitriptyline induce alterations in intestinal microbiota and gut microbiome function in rats exposed to chronic unpredictable mild stress
Source: Transl Psychiatry. 2021 Feb 18;11:131. doi: 10.1038/s41398-021-01254-5 (PMC7892574; doi:10.1038/s41398-021-01254-5)
Supplement: Supplementary file 10 — Supplementary Figure 9 [file 41398_2021_1254_MOESM10_ESM.pdf]

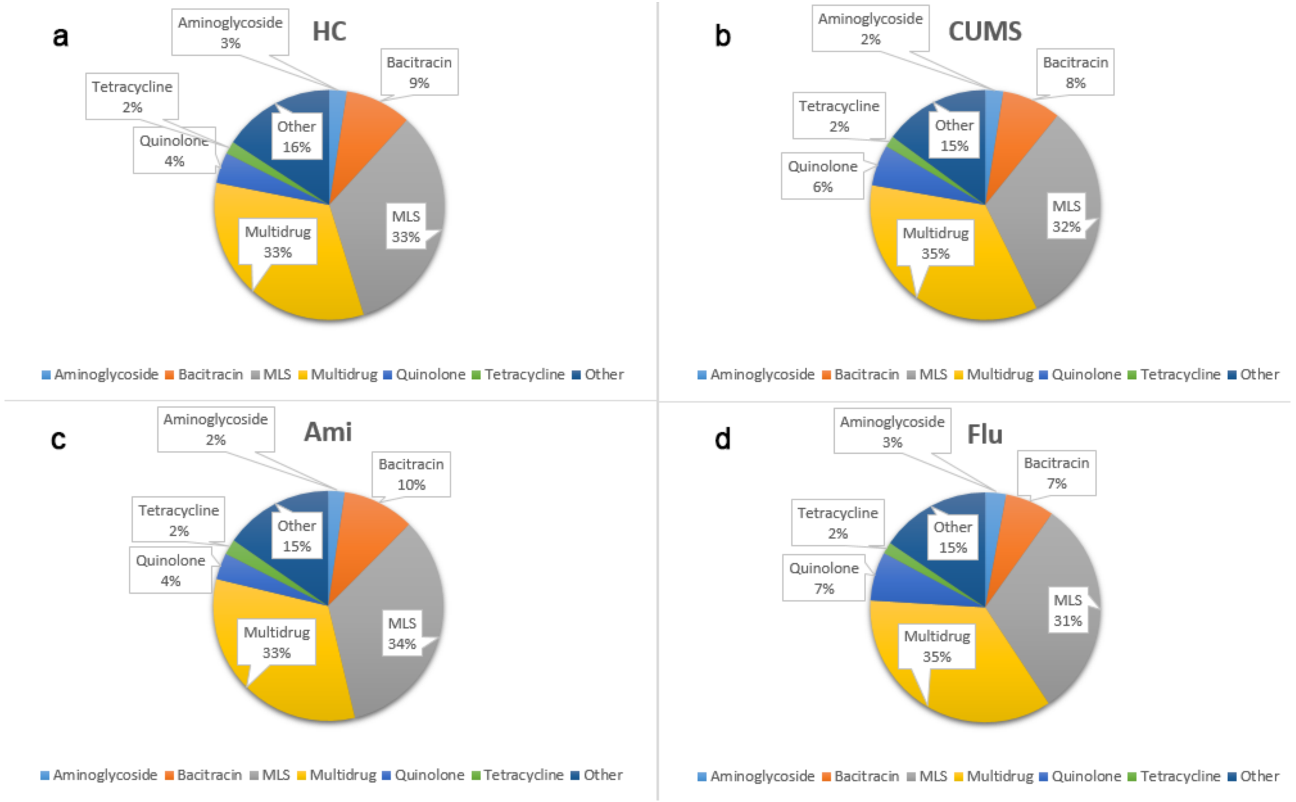
a
HC
Aminoglycoside 3%
Bacitracin 9%
Tetracycline 2%
Quinolone 4%
Other 16%
MLS 33%
Multidrug 33%
Aminoglycoside Bacitracin MLS Multidrug Quinolone Tetracycline Other
b
CUMS
Aminoglycoside 2%
Bacitracin 8%
Tetracycline 2%
Quinolone 6%
Other 15%
MLS 32%
Multidrug 35%
Aminoglycoside Bacitracin MLS Multidrug Quinolone Tetracycline Other
c
Ami
Aminoglycoside 2%
Bacitracin 10%
Tetracycline 2%
Quinolone 4%
Other 15%
MLS 34%
Multidrug 33%
Aminoglycoside Bacitracin MLS Multidrug Quinolone Tetracycline Other
d
Flu
Aminoglycoside 3%
Bacitracin 7%
Tetracycline 2%
Quinolone 7%
Other 15%
MLS 31%
Multidrug 35%
Aminoglycoside Bacitracin MLS Multidrug Quinolone Tetracycline Other
