# Supplementary material for: Antidepressants fluoxetine and amitriptyline induce alterations in intestinal microbiota and gut microbiome function in rats exposed to chronic unpredictable mild stress
Source: Transl Psychiatry. 2021 Feb 18;11:131. doi: 10.1038/s41398-021-01254-5 (PMC7892574; doi:10.1038/s41398-021-01254-5)
Supplement: Supplementary file 13 — Supplementary Table 1 [file 41398_2021_1254_MOESM13_ESM.docx]

**Supplementary Table 1：**Permutational multivariate analysis of variance (PERMANOVA) tests of the bacterial fecal microbiota on the weighted and unweighted UniFrac distances between HC and groups at week 9.

|  | Metric | F | R^2^ | *P-Value* |
| --- | --- | --- | --- | --- |
| HC (n=12)  vs.  CUMS (n=12) | Weighted Unifrac | 4.41 | 0.17 | 0.011 |
|  | Unweighted Unifrac | 7.05 | 0.24 | 0.001 |

HC, healthy control rats; CUMS, chronic unpredictable mild stress rats
