# Supplementary material for: Antidepressants fluoxetine and amitriptyline induce alterations in intestinal microbiota and gut microbiome function in rats exposed to chronic unpredictable mild stress
Source: Transl Psychiatry. 2021 Feb 18;11:131. doi: 10.1038/s41398-021-01254-5 (PMC7892574; doi:10.1038/s41398-021-01254-5)
Supplement: Supplementary file 14 — Supplementary Table 2 [file 41398_2021_1254_MOESM14_ESM.docx]

**Supplementary Table 2：**Permutational multivariate analysis of variance (PERMANOVA) tests of the bacterial fecal microbiota on the weighted and unweighted UniFrac distances in HC, CUMS, Ami and Flu groups.

|  | **Metric** | **F** | **R^2^** | ***P-value*** |
| --- | --- | --- | --- | --- |
| HC (n=12)  vs  CUMS (n=6) | Weighted UniFrac | 0.86 | 0.05 | 0.004 |
|  | Unweighted UniFrac | 1.97 | 0.10 | 0.001 |
| CUMS (n=6)  vs  Ami (n=6) | Weighted UniFrac | 2.08 | 0.17 | 0.01 |
|  | Unweighted UniFrac | 2.08 | 0.17 | 0.005 |
| CUMS (n=6)  vs  Flu (n=7) | Weighted UniFrac | 8.14 | 0.42 | 0.002 |
|  | Unweighted UniFrac | 2.05 | 0.15 | 0.002 |
| HC (n=12)  vs  Ami (n=6) | Weighted UniFrac | 1.51 | 0.08 | 0.19 |
|  | Unweighted UniFrac | 2.01 | 0.11 | 0.001 |
| HC (n=12)  vs  Flu (n=7) | Weighted UniFrac | 6.41 | 0.27 | 0.005 |
|  | Unweighted UniFrac | 2.21 | 0.11 | 0.001 |

HC, healthy control rats; CUMS, chronic unpredictable mild stress rats; Ami, amitriptyline treatment rats; Flu, fluoxetine hydrochloride treatment rats.
