# Supplementary material for: Antidepressants fluoxetine and amitriptyline induce alterations in intestinal microbiota and gut microbiome function in rats exposed to chronic unpredictable mild stress
Source: Transl Psychiatry. 2021 Feb 18;11:131. doi: 10.1038/s41398-021-01254-5 (PMC7892574; doi:10.1038/s41398-021-01254-5)
Supplement: Supplementary file 15 — Supplementary Table 3 [file 41398_2021_1254_MOESM15_ESM.docx]

**Supplementary Table 3：**Relative abundance of the most common OTUs on phylum and family levels from the different groups.

| **Species name** | **Mean ± Sd (%)** | | | | ***p*-value** | | | | |
| --- | --- | --- | --- | --- | --- | --- | --- | --- | --- |
|  | **HC** | **CUMS** | **Flu** | **Ami** | **CUMS**  ***vs* HC** | **Flu**  ***vs* HC** | **Ami**  ***vs* HC** | **Flu *vs***  **CUMS** | **Ami *vs***  **CUMS** |
| *p__Firmicutes* | 68.97 ± 6.302 | 71.45 ± 6.835 | 59.44 ± 5.671 | 63.93 ± 9.281 | 0.915 | 0.05948 | 0.5564 | **0.03754*** | 0.3358 |
| *p__Bacteroidetes* | 26.99 ± 5.973 | 24.48 ± 5.974 | 37.19 ± 5.44 | 31.77 ± 7.67 | 0.8834 | **0.01821*** | 0.5106 | **0.01094*** | 0.2712 |
| *p__Actinobacteria* | 2.385 ± 1.613 | 1.933 ± 0.787 | 1.229 ± 0.260 | 1.356 ± 0.507 | 0.8815 | 0.2144 | 0.3503 | 0.7322 | 0.8471 |
| *p__Tenericutes* | 1.306 ± 0.666 | 1.585 ± 1.423 | 1.743 ± 1.302 | 1.340 ± 1.224 | 0.9672 | 0.8729 | 0.9999 | 0.8729 | 0.9851 |
| *f__Bacteroidales_*S24-7_group | 22.33 ± 5.624 | 19.95 ± 4.968 | 28.64 ± 7.203 | 23.23 ± 4.454 | 0.8738 | 0.1716 | 0.9917 | 0.0816 | 0.8041 |
| *f__Lactobacillaceae* | 26.49 ± 12.70 | 27.38 ± 10.01 | 13.74 ± 7.305 | 19.50 ± 11.51 | 0.9988 | 0.1447 | 0.6590 | 0.2043 | 0.6757 |
| *f__Lachnospiraceae* | 19.34 ± 7.116 | 17.41 ± 2.092 | 23.93 ± 7.442 | 21.69 ±5.443 | 0.9435 | 0.5093 | 0.9039 | 0.3406 | 0.7078 |
| *f__Ruminococcaceae* | 14.04 ± 3.103 | 15.16 ± 2.956 | 13.78 ± 2.211 | 13.67 ± 2.061 | 0.8778 | 0.9978 | 0.9946 | 0.8417 | 0.8255 |
| *f__Peptostreptococcaceae* | 6.497 ± 3.586 | 8.121 ± 5.074 | 5.151 ± 4.684 | 5.254 ± 2.695 | 0.8839 | 0.9193 | 0.9433 | 0.6298 | 0.6811 |
| *f__Bacteroidaceae* | 2.482 ± 0.7548 | 2.069 ± 0.6582 | 4.753 ± 2.699 | 6.065 ± 4.200 | 0.9876 | 0.2469 | **0.0356*** | 0.2389 | **0.0446*** |
| *f__Prevotellaceae* | 1.667 ± 0.8154 | 2.049 ± 1.451 | 3.073 ± 2.702 | 1.649 ± 0.9135 | 0.9704 | 0.3303 | 0.9999 | 0.7103 | 0.9776 |
| *f__Coriobacteriaceae* | 2.038 ± 1.466 | 1.712 ± 0.7152 | 1.061 ± 0.249 | 1.714 ± 0.5306 | 0.9372 | 0.2768 | 0.4255 | 0.7259 | 0.8398 |
| *f__norank_o__Mollicutes_*RF*9* | 1.304 ± 0.6668 | 1.58 ± 1.424 | 1.740 ± 1.301 | 1.336 ± 1.224 | 0.9682 | 0.8736 | 0.9999 | 0.9952 | 0.9852 |
| *f__Erysipelotrichaceae* | 0.8449 ± 0.4246 | 1.468 ± 1.158 | 1.256 ± 0.406 | 1.312 ± 0.5919 | 0.3206 | 0.6280 | 0.5678 | 0.9510 | 0.9815 |
| *f__Clostridiaceae_1* | 0.3628 ± 0.2108 | 0.704 ± 0.1918 | 0.4185 ± 0.2311 | 1.151 ± 0.673 | 0.2991 | 0.9899 | **0.0013**** | 0.5445 | 0.2000 |
| *f__Porphyromonadaceae* | 0.3792 ± 0.07 | 0.3515 ± 0.09 | 0.57 ± 0.14 | 0.7004 ± 0.15 | 0.968 | **0.011*** | **5.41E-05***** | **0.014**** | **0.0001***** |

HC, healthy control rats; CUMS, chronic unpredictable mild stress rats; Ami, amitriptyline treatment rats; Flu, fluoxetine hydrochloride treatment rats. Statistical analysis was performed by the Kruskal-Wallis H test. **p* < 0.05, ** *p* < 0.01, *** *p* < 0.001.
