# Supplementary material for: Antidepressants fluoxetine and amitriptyline induce alterations in intestinal microbiota and gut microbiome function in rats exposed to chronic unpredictable mild stress
Source: Transl Psychiatry. 2021 Feb 18;11:131. doi: 10.1038/s41398-021-01254-5 (PMC7892574; doi:10.1038/s41398-021-01254-5)
Supplement: Supplementary file 16 — Supplementary Table 4 [file 41398_2021_1254_MOESM16_ESM.docx]

**Supplementary Table 4 ：**Percentage of ARGs in different groups.

| ARGs | Relative abundance/ % | | |  | |  |  | |
| --- | --- | --- | --- | --- | --- | --- | --- | --- |
|  | | HC | CUMS | | Ami | | | Flu |
| *aph3iii*A | | 27.34±3.35 | 18.61±2.44 | | 27.22±4.02 | | | 28.19±7.44 |
| *bac*A | | 14.37±0.73 | 15.64±2.77 | | 15.74±2.90 | | | 17.17±1.16 |
| *tet*Q | | 9.60±0.21 | 13.32±7.23 | | 6.52±1.50 | | | 13.68±5.19 |
| *tet*W | | 9.55±1.59 | 10.64±4.39 | | 8.98±3.04 | | | 10.87±3.60 |
| *tet*O | | 8.16±1.70 | 10.41±5.57 | | 11.45±4.50 | | | 3.85±1.83 |
| *erm*B | | 9.58±1.84 | 7.85±4.32 | | 8.08±2.33 | | | 7.06±3.71 |
| *tet*40 | | 4.49±0.46 | 4.52±3.07 | | 5.39±1.34 | | | 4.08±0.76 |
| *tet*32 | | 4.34±0.34 | 2.46±1.18 | | 3.94±0.98 | | | 3.05±1.76 |
| *erm*F | | 3.73±0.41 | 4.03±1.85 | | 2.281±1.33 | | | 3.68±1.33 |
| *ant6i*A | | 2.40±0.18 | 2.16±0.38 | | 2.49±1.13 | | | 2.00±0.87 |
| *erm*T | | 2.26±1.37 | 3.74±5.62 | | 0.67±0.25 | | | 0.43±0.45 |
| *erm*G | | 0.31±0.01 | 0.69±0.22 | | 0.86±0.92 | | | 1.25±0.45 |
| *aad*D | | 0.45±0.19 | 0.63±0.69 | | 0.78±1.13 | | | 0.91±1.38 |
| *lnuA* | | 0.28±0.19 | 0.99±1.51 | | 0.18±0.16 | | | 0.19±0.25 |
| *mef*A | | 0.20±0.08 | 0.73±0.98 | | 0.22±0.11 | | | 0.31±0.36 |

HC, healthy control rats; CUMS, chronic unpredictable mild stress rats; Ami, amitriptyline treatment rats; Flu, fluoxetine hydrochloride treatment rats.
